# Hoberman‐Inspired Reconfigurable DNA Origami Ring for Nanoscale Capture

**DOI:** 10.1002/smll.75370

**Published:** 2026-08-25

**Authors:** Henry Wisniewski, Minu Saji, Moisés Reyes, Qi Yang, Fei Zhang

**Affiliations:** ^1^ Department of Chemistry Rutgers University, New Jersey Newark USA

**Keywords:** colloidal gold, DNA nanotechnology, DNA origami, dynamic nanostructure, Holliday junction, nanorobotics

## Abstract

Dynamic DNA origami systems have enabled programmable nanoscale motion and target‐responsive structural transformations for applications in molecular capture, sensing, and nanorobotics. However, achieving programmable interactions with targets of varying sizes and morphologies remains challenging for many dynamic DNA nanostructures. Here, we present a reconfigurable DNA origami ring inspired by Hoberman mechanisms by translating scissor‐linkage architectural principles into a nanoscale DNA system. The DNA ring features right‐angled arms connected via rotational Holliday junctions to form radially arranged scissor‐mechanism linkages capable of cooperative motion. Coordinated rotation of the scissor units enables reversible contraction and expansion of the ring, producing three conformational states: closed, neutral, and open. Conformational switching between states is achieved using strand displacement. By functionalizing the ring with capture probes, conformation‐dependent organization of gold nanoparticles is demonstrated. Additionally, the ring successfully captures three DNA nanostructure tiles with distinct sizes and morphologies and exhibits conformation‐dependent binding preferences. This work presents a mechanically coordinated DNA origami architecture for programmable nanoscale organization and molecular capture.

## Graphical abstract

A Hoberman‐inspired DNA origami ring with coupled scissor‐like motion is designed and assembled. Reversible reconfiguration produces different conformations for organization of gold nanoparticles and capture of DNA nanostructures with varied sizes and geometries.

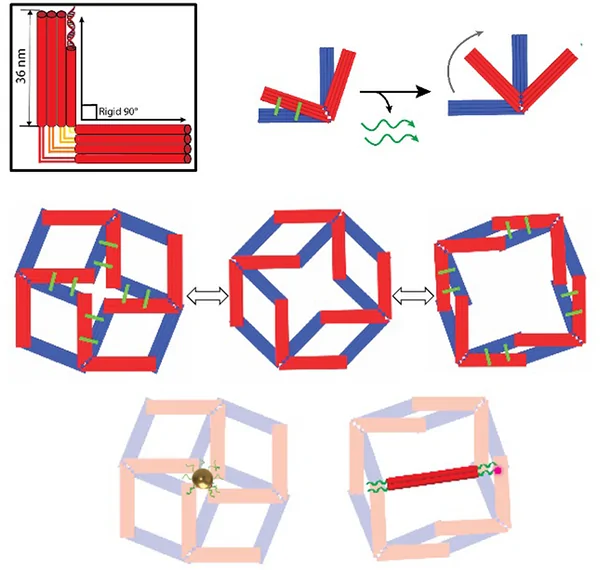

## 1 Introduction

DNA origami has emerged as a powerful technique to create DNA nanostructures and nanodevices for applications in biosensing, nanofabrication, and biomedicine [1, 2, 3, 4, 5, 6]. Beyond static assemblies, increasing efforts have focused on dynamic DNA nanostructures, enabling more complex systems such as DNA payloads capable of controllable release of drug molecules, multi‐stage and multi‐input logic gates for DNA‐based circuits, and DNA tweezers that can capture and detect specific species [7, 8, 9, 10, 11]. In particular, dynamic DNA origami nanostructures capable of interacting with proteins, nanoparticles, and other nanoscale species have attracted broad interest because of their programmability and structural precision [12, 13, 14, 15, 16, 17, 18]. However, interactions with targets of different sizes, shapes, and morphologies remain challenging for many dynamic DNA nanostructures as most systems are designed with localized mechanical motion. Developing DNA nanostructures capable of coordinated and tunable large‐scale transformations would therefore expand the applications of dynamic DNA origami systems for programmable molecular capture, nanoscale assembly, and mechanically responsive nanodevices.

Hoberman mechanisms are a class of transformable architectures that utilize interconnected scissor linkages to enable coordinated expansion and contraction while maintaining overall structural integrity [19, 20]. These systems achieve synchronized motion through mechanically coupled rotational joints that propagate structural reconfiguration across the entire architecture. Such deployable mechanisms enable structures to transform between two or more conformational states, allowing a single structure to adapt to different environments or purposes, especially where accessibility is difficult, such as in outer space or inside the human body. Translating Hoberman principles to DNA nanotechnology enables the development of mechanically coupled nanostructures with tunable geometries and functions.

In this work, a transformable, Hoberman‐inspired DNA origami ring is presented. The structure consists of right‐angled arm units connected via rotating Holliday junctions to form radially arranged scissor mechanisms that enable uniform, coupled motion. Introduction of different sets of locking strands at specific vertices stabilized the ring in a closed or open conformation, while the absence of locking strands released the origami ring into a freely dynamic, neutral state. Reconfiguration between conformations is achieved through toehold‐mediated strand‐displacement reactions (SDR). Finally, the resulting mechanically coordinated DNA origami architecture was further functionalized for conformation‐dependent organization of 5 nm gold nanoparticles and capture of three DNA nanostructures of varying size, shape, and morphology. This work introduces a transformable DNA origami platform that expands the dynamic and functional capabilities of nanoscale self‐assembled systems.

## 2 Results

The DNA ring was formed using the DNA origami technique via folding a DNA scaffold, M13mp18, by hundreds of complementary staple strands (Figure 1A). The overall origami ring was designed approximately 72 *nm* in diameter and 4 *nm* thick (Figure 1B), where two layers of DNA 4‐helix bundle (4‐by‐1) were arranged carefully in an interlocked way using the square lattice layout in CaDNAno. The alternating interlocking is achieved by positioning the Holliday junctions at specific locations along the scaffold. Because of the helical twist of DNA, these junctions are oriented to face either upward or downward, enabling connections between the upper and lower layers. The top and bottom layers were linked by eight Holliday junctions (Figure 1C), allowing free rotation between layers at each vertex. The ring contained eight arm units (Figure 1D) that formed a 90^°^ angle. Two arm units were then stacked and connected via a Holliday junction near the vertex to form a scissor unit (Figure 1E). Four scissor units were further connected radially using eight Holliday junctions at the ends of arms. This construct gave the DNA ring 4‐fold symmetry and a reconfigurable frame (Figure 1F). By rotating the ring inward or outward, a closed or open conformation could be achieved (Figure 1G). The scaffold routing (Figure 1H) reflected the 4‐fold symmetry by the presence of four repeating scaffold blocks. 90^°^ angles were positioned at the center of each repeating block (see Figure S1 for more details). The 90^°^ angle was created by arranging increasing lengths of DNA helices from inside to outside [21] to create a right‐angle (Figure 1I,J). The arm angles were measured giving an average angle of 89.07 degrees (SD = 14.96), validating the right‐angle design (Figure S3). Finally, the blunt ends of each DNA scissor unit had single‐stranded hangovers to prevent undesired stacking interactions (Figure 1K).

**FIGURE 1:**
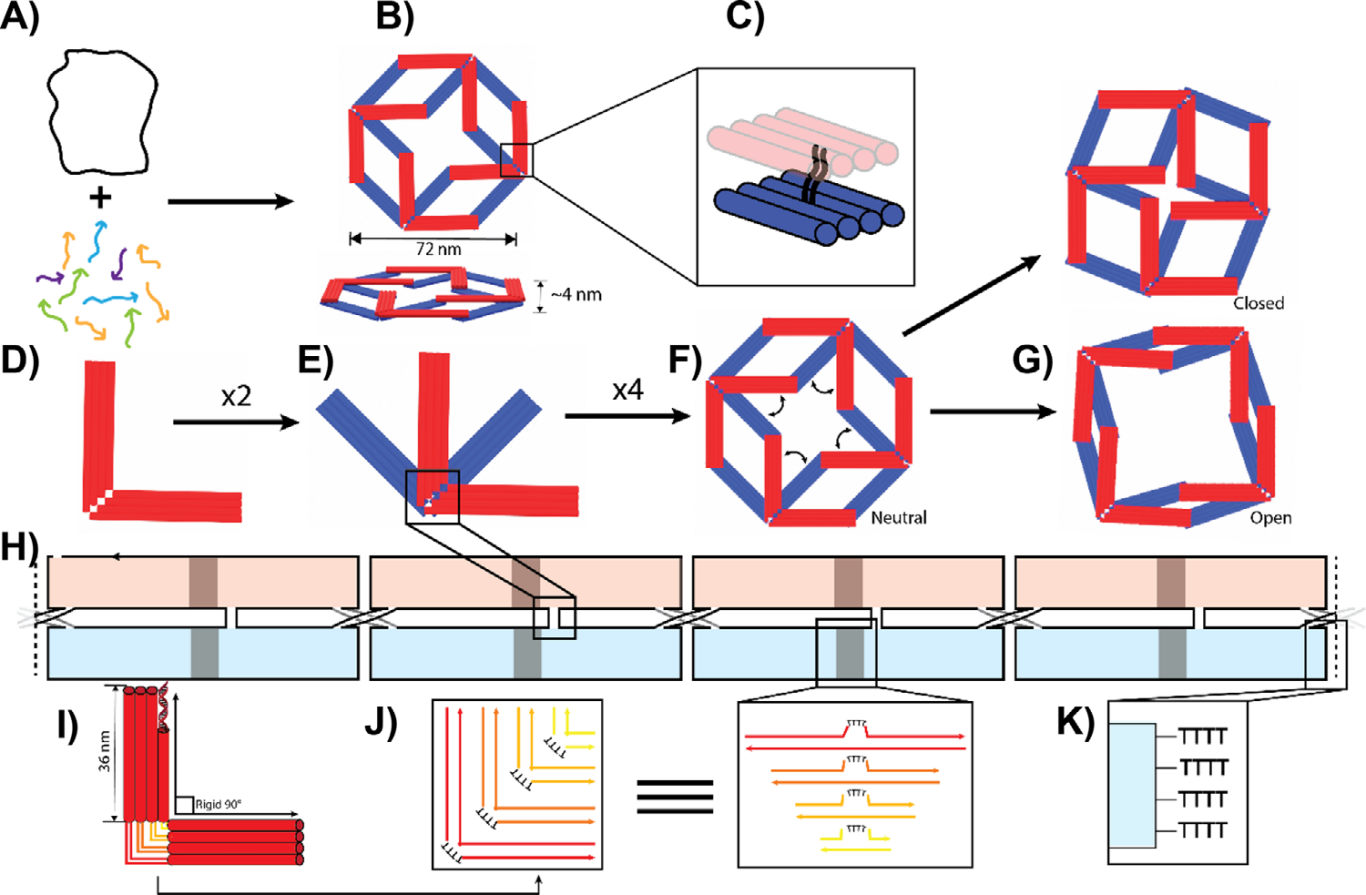
Design schematics of the DNA origami Hoberman ring. (A) M13mp18 DNA scaffold and complementary DNA staples for DNA origami formation. (B) Model of the DNA Hoberman ring. (C) Schematic of a rotational Holliday junction connecting the two DNA layers. (D) Single arm unit. (E) A scissor unit formed by connecting and layering two arm units at the vertex via a Holliday junction. (F) Fully formed ring composed of four radially arranged scissor units, enabling coordinated rotational motion. (G) Closed and open conformations generated from rotating the ring inward or outward. (H) Scaffold routing diagram showing top layer (red) and lower layer (blue) with Holliday junctions and 90^°^ angle (shaded regions). Dashed lines indicate connectivity to form the single loop scaffold. (I) 90^°^ angle design schematic. (J) Zoomed routing of 90^°^ angle and linearized showing nested truncation). (K) 4T motif hangovers to prevent blunt end stacking interactions.

To control the configurations of the ring, locking strands were employed to pull either the inner angles (Figure 2A) or outer angles (Figure 2B). Depending on which vertices were constrained, the ring adopted a closed or open conformation, while the absence of locking strands produced the freely dynamic neutral state (Figure 2C). Atomic force microscopy (AFM) was used to characterize each conformation (Figures S4–S6), confirming the successful formation of all three states. To further quantify these conformations, a quadrilateral was inscribed by connecting the four most inner points of each ring using ImageJ (Figure 2D). The inner areas of the quadrilaterals were measured, yielding average values of 375.2 nm^2^ (sd = 80.56), 722.9 nm^2^ (sd = 225.40), and 1394.5 nm^2^ (sd = 234.70) for the closed, neutral, and open conformations respectively. These differences were statistically significant, validating inner area as a metric for conformation classification. To evaluate assembly yields, each conformation was independently annealed and analyzed by AFM. Individual rings were classified as closed, neutral, or open based on their measured inner areas relative to the established average value, and the distributions for each conformation were plotted (Figure 2E and Figure S7). As expected, the dominant conformation of closed and open rings corresponded to the intended annealed state, where the closed ring showed 97.14% and only 2.86% as neutral, and, in the open configuration, 91.74% of the rings were classified as open and 8.26% as neutral. In contrast, the neutral conformation had a wider distribution with 71.31% as neutral, 21.31% as closed, and 7.38% as open. The greater distribution is consistent with the greater rotational freedom of the unconstrained neutral conformation, allowing a wider range of accessible geometries. Each conformation was also characterized by agarose gel electrophoresis, which showed minimal mobility differences among the three states (Figure S8).

**FIGURE 2:**
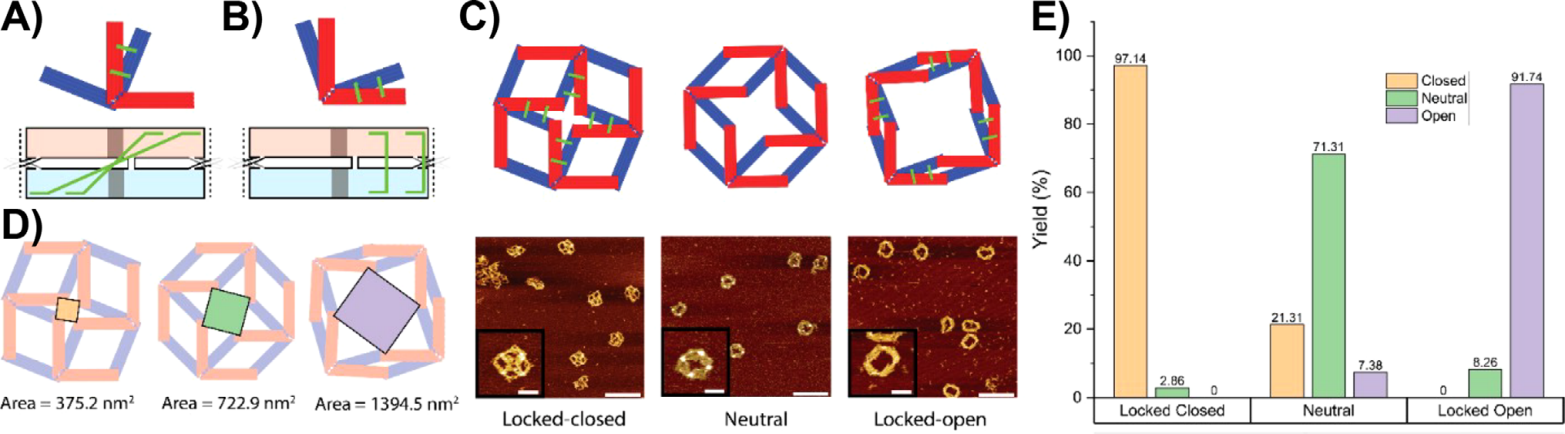
Conformational control and structural characterization of the DNA origami ring. (A) Schematic of inner locking strands (green) with model (top) and strand routing (bottom). (B) Schematic of outer locking strands (green) with model (top) and strand routing (bottom). (C) Model showing configuration of locking strands (green) of the three conformations, the closed, neutral, and open (left to right) (top) and AFM of all three conformations (bottom). Scale bar = 200 nm, zoomed scale bar = 50 nm. (D) Diagram showing transcribed quadrilaterals for each conformation used to calculate area for each conformation. Quadrilateral colors reflect closed (orange), neutral (green), and open (purple) conformations. (E) Distribution of conformational populations for each conformation. Conformations tallied via classification using the nearest, average quadrilateral areas (N = 70, 122, and 114 for closed, neutral, and open conformations respectively).

To investigate the dynamic reconfiguration of the Hoberman‐inspired ring, conformational switching between locked states was performed. To reconfigure the ring, the initial locking strands needed to be released from the origami to restore rotational freedom. Then, the new locking strands for the final conformation were introduced to re‐lock the ring to the target conformation (Figure 3A). This was achieved through toehold‐mediated strand‐displacement reactions, in which each locking strand was extended with a 6‐nt toehold (Figure 3B). Reconfigurations from closed‐to‐open and open‐to‐closed were performed. AFM imaging was conducted before and after each transition (Figure 3C,D and Figures S9 and S10). The conformational distributions were quantified using the same inner‐area classification method described above.

**FIGURE 3:**
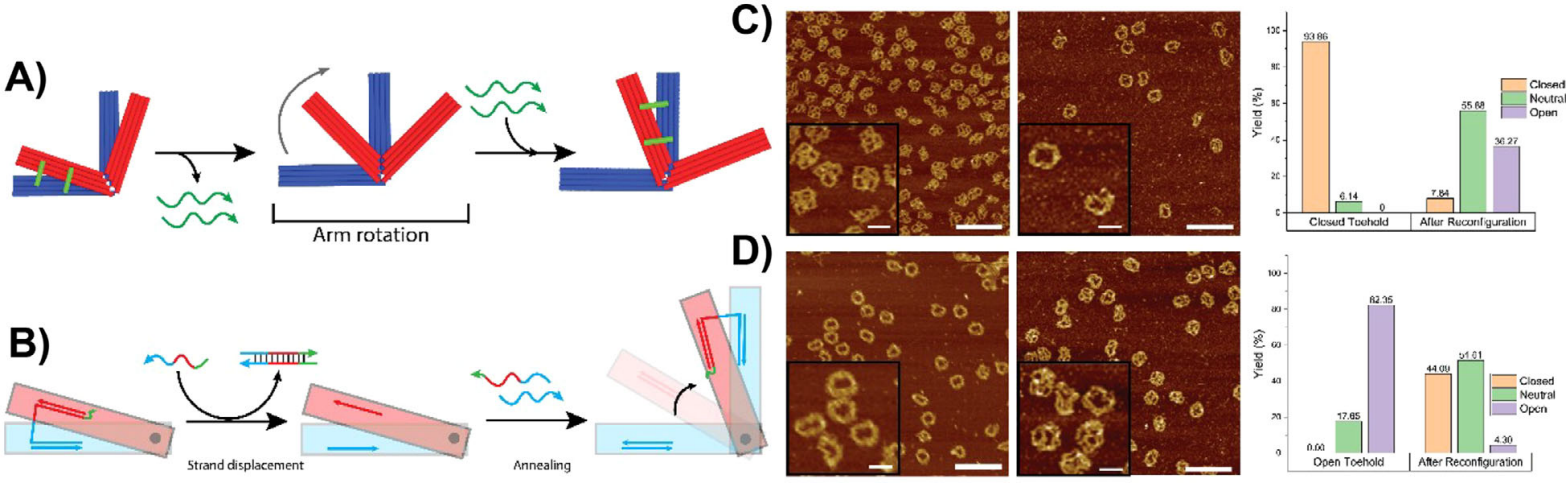
Strand‐displacement‐mediated reconfiguration of the DNA origami ring. (A) Schematic of the release, rotation, and relocking of the arms. (B) Schematic showing how strand displacement was implemented to enable reconfiguration. (C) AFM of the closed ring before reconfiguration (left) and after reconfiguration to open conformation (right). Large scale bar = 200 nm, zoomed‐in scale bar = 50 nm. Ring conformation distributions before and after reconfiguration. (D) AFM images of the open ring before (N = 114) reconfiguration (left) and after (N = 102) reconfiguration to closed conformation (right). Large‐scale bar = 200 nm, zoomed‐in scale bar = 50 nm. Ring conformation distributions before (N = 129) and after reconfiguration (N = 93).

For the closed‐to‐open reconfiguration, the percentage of the closed conformation dropped from 93.86% to 7.84%, representing a 91.65% decrease, and the population of the open conformation increased from 0% to 36.27%. Although this is lower than the 82.35% yield obtained by direct annealing of the open conformation, it demonstrates the capability of reconfiguration to the target state. Similarly, during the open‐to‐closed reconfiguration, the percentage of the open conformation decreased from 82.35% to 4.30%, corresponding to a 94.78% reduction, while the closed conformation increased to 44.09%, compared with the 91.65% yield achieved by direct annealing. In both transitions, the neutral conformation remained the dominant population, accounting for approximately 51%–55% of the structures, slightly exceeding the population of the target conformation. These results confirm that toehold‐mediated strand displacement effectively releases the initial locking strands and enables reconfiguration between the two extreme conformations. However, around half of the rings remain in the neutral state after re‐locking. The open‐to‐closed reconfiguration exhibited slightly higher efficiency than the closed‐to‐open transition. We attribute this difference to the higher inter‐helical repulsion in the open conformation, where the arm bundles are concentrated into closely spaced pairs along the ring perimeter. The more evenly distributed helices in the closed conformation are likely energetically more favorable.

The control experiment of using only two pairs of locking strands, instead of four, demonstrated that only two opposing locking sites are sufficient to stabilize either the open or closed conformation, supporting the mechanically cooperative behavior of the Hoberman‐inspired architecture (Figure S11). Furthermore, reconfiguration from the preassembled neutral state produced comparable or lower yields than strand‐displacement‐mediated reconfiguration (Figure S12). These results demonstrate that the Hoberman‐inspired ring is dynamically reconfigurable while highlighting the challenge of achieving complete switching in a mechanically coupled DNA architecture.

To demonstrate the function of the transformable ring as a nanoscale capture platform, we first examined conformation‐dependent organization of 5 nm gold nanoparticles. The gold nanoparticle surface was functionalized using thiol‐modified DNA (Figure 4A), while complementary capture probes were added to the inner region of the ring (Figure 4B). Because the spatial arrangement of these probes depends on ring conformation, different capture geometries were expected. In the closed state, the closely packed probes were designed to promote centralized nanoparticle capture, whereas the increased spacing in the neutral and open conformations was expected to permit multiple, spatially separated attachment sites (Figure 4C).

**FIGURE 4:**
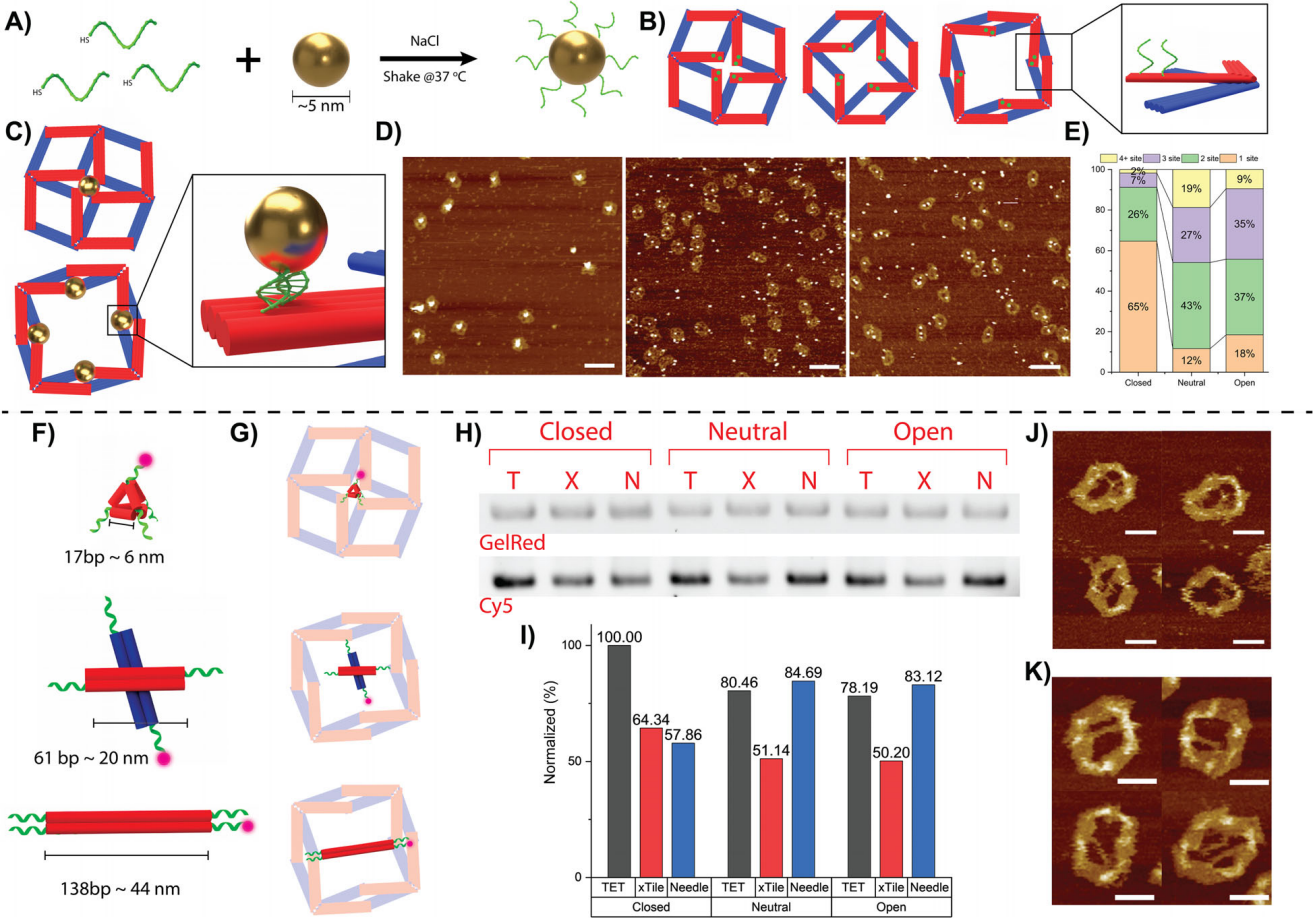
Functional applications of the ring by cargo organization and nanoscale capture. (A) Gold nanoparticle scheme showing thiol‐modified DNA reacting with gold nanoparticles to cover gold nanoparticle with DNA. (B) Locational layout of capture probes (green) for the closed, neutral, and open ring conformations. A zoomed‐in view shows how each probe pair extrudes upward for better attachment efficiency. (C) Examples of gold nanoparticle attachment on the closed (upper) and open (lower) conformations. Zoomed view shows how DNA‐functionalized gold nanoparticles attaches to the capture probes on ring via hybridization. (D) AFM of the gold nanoparticle attachment for the closed (Left), neutral (middle), and open (right) conformations. Scale bar = 200 nm. (E) Distribution of the three conformations and how many discrete gold attachment sites visible from AFM data. (N = 170, 155, and 190 for the closed, neutral, and open conformations, respectively). (F) Tetrahedron, xTile, and Needle DNA nanostructures captured by the ring. Each nanostructure was modified with anchor probes (green) and Cy5‐modified DNA (pink circle represents Cy5 molecule). Approximate dimensions are given. (G) Scaled depiction of most ideal conformations to capture each nanostructure based on nanostructure size. (H) Native agarose gel of the tetrahedron (lanes T), xTile (lanes X), and needle (lanes N) nanostructures binding to each conformation of the ring. The top shows only the GelRed signal, and the bottom shows only the Cy5 signal. (I) Normalized band volumes from the native agarose Cy5 signal. (J) AFM images of the xTile binding to the open ring. Scale bar = 50 nm. (K) AFM images of the needle binding to the open ring. Scale bar = 50 nm.

AFM imaging confirmed this conformation‐dependent behavior (Figure 4D and Figure S13). In the closed conformation, nanoparticles predominantly localized as a single bright center within the ring, consistent with efficient capture. In contrast, the neutral and open gold states exhibited more distributed attachment patterns, with multiple nanoparticle binding sites becoming increasingly prevalent as probe spacing increased (Figures S14 and S15). Quantitative analysis supported this trend (Figure 4E and Figure S16), with 1‐site occupation in the closed state (65%), while neutral and open conformations showed a dramatic drop in 1‐site attachment to 12% and 18%, respectively. Instead, there was more prominent multi‐site attachment with 43% and 37% 2‐site attachment, and 27% and 35% 3‐site attachment for the neutral and open conformation, respectively. These results demonstrate that ring geometry can be used to spatially organize nanoscale cargo in a conformation‐dependent manner.

To estimate the number of AuNPs captured within the closed ring, free functionalized AuNPs were analyzed by measuring the area of background, unattached AuNPs, resulting in an average of 281.5 nm^2^ (SD = 64.6, N = 100). The areas of AuNP clusters located at the center of closed rings were then measured, resulting in an average area of 391.19 nm^2^ (SD = 107.3, N = 94). By comparing the average projected areas of attached and free AuNPs, the average occupancy was estimated to be 1.39 AuNPs per ring. This result indicates that the closed conformation predominantly captures a single AuNP within its central cavity. Furthermore, the measured area of free functionalized AuNPs agreed well with the theoretical number of 253.1 nm^2^, supporting the validity of the area‐based occupancy analysis.

To further investigate the ability of the ring to capture DNA nanostructures with distinct geometries, three structurally diverse DNA nanostructures were selected as the targets (Figure 4F), including a tetrahedron (TET), a two‐layered X‐tile (xTile), and a needle‐like structure (Needle) [22, 23]. Each nanostructure was functionalized with anchor strands and Cy5 labels, allowing easy distinction between the DNA origami and the DNA nanostructures. The nanostructures were bound to the origami via a glue strand, which hybridized both the nanostructure anchor strand and DNA origami capture strands (Figure S19). Based on the relative sizes of the nanostructures, the compact TET was expected to fit within a closed conformation ring, while the larger xTile and Needle would require more expanded geometries of an open conformation ring (Figure 4G).

Rather than pre‐locking the ring into specific conformations, these experiments were performed using the neutral state to assess whether the unconstrained architecture could dynamically adapt to different cargo geometries. Native agarose gel electrophoresis showed successful formation of the DNA origami ring and retention of assembled complexes (Figure 4H and Figure S20). While GelRed imaging showed minimal band shifts between the origami ring and cargo‐bound origami, Cy5 fluorescence revealed clear co‐migration of all three nanostructures with the origami band, confirming successful capture. AFM imaging further verified the association of the DNA nanostructures with the ring in open or neutral configuration (Figure 4J,K and Figures S21–S23, S25 and S26). Analysis of the conformational distributions showed that the neutral conformation remained the predominant population for all three nanostructures (Figure S24), indicating that large‐scale reconfiguration is not required for cargo binding. Nevertheless, the tetrahedron and xTile samples exhibited modest increases in the closed population, whereas the needle sample showed a higher proportion of the open conformation. These trends are consistent with the relative sizes and geometries of the nanostructures, suggesting that cargo geometry can impact the conformational distribution of the mechanically flexible ring. These results indicate that the neutral conformation is sufficient to capture structurally distinct DNA nanostructures, while the binding behavior is influenced by the probe number, probe distribution, and geometric match between the target cargo and the capture ring.

## 3 Discussion

In this work, we demonstrated a dynamic and functional DNA origami ring inspired by Hoberman linkage mechanics. While many dynamic DNA nanostructures reconfigure through localized conformational changes, this design introduces a mechanically coupled architecture in which coordinated radial motion arises from interconnected scissor units. By selectively locking specific regions of the origami, the ring could be stabilized in closed and open conformations, whereas the absence of locking strands yielded a freely dynamic neutral state. Reconfiguration between states demonstrated the coordination nature of the Hoberman DNA ring. Toehold‐mediated strand displacement effectively released the initial locking constraints, enabling reconfiguration between the two extreme conformations. Although both target conformations were regenerated after reconfiguration, the neutral conformation remained the predominant population, indicating that complete switching within this architecture remains challenging. Control experiments further showed that two opposing locking sites were sufficient to stabilize either the open or closed conformation. These results establish the Hoberman‐inspired ring as a DNA nanostructure capable of reversible large‐scale reconfiguration.

By functionalizing the ring with capture probes, we demonstrated conformation‐dependent organization of gold nanoparticles, with the closed conformation predominantly capturing a single nanoparticle within its central cavity and the more expanded conformations producing multiple spatially separated attachment sites. We further showed that the same platform could capture DNA nanostructures with different sizes and geometries. AFM imaging and gel electrophoresis confirmed successful binding of all three nanostructures, while conformational analysis revealed that the neutral state remained the dominant population after binding. Rather than requiring large‐scale adaptive reconfiguration, these results indicate that the mechanically flexible neutral conformation is sufficient for cargo capture. At the same time, modest shifts in the conformational distributions, together with differences in binding efficiency among the three conformations, highlight the influence of ring geometry, probe distribution, and target geometry on the binding interaction. These results show that the Hoberman‐inspired ring can organize nanoscale cargo and capture DNA nanostructures with different geometries.

While the current design demonstrates coordinated DNA origami for target capture, several opportunities remain for further development. Improved strategies for synchronized actuation will likely be required to achieve efficient full‐state switching between extreme conformations. For example, stimuli‐responsive motifs, such as G‐quadruplexes, i‐motifs, or photo‐responsive azobenzene‐modified DNA, could be incorporated to enable autonomous reconfiguration in response to ions, pH, or light [24, 25]. Such capabilities could enable programmable recognition and capture of biological targets with different sizes and geometries, including extracellular vesicles, viral particles, and protein assemblies. Additionally, other functional elements could be integrated to expand the transformation mechanisms, such as fluorescent‐quenching dyes or fluorescent split‐aptamers, as reporting signals for biosensing and detection [26]. Beyond molecular capture, the mechanically coupled architecture could serve as a programmable nanoreactor in which structural changes regulate the proximity of multiple reactants, or as a multivalent scaffold for controlling ligand presentation and receptor clustering. Furthermore, the ring's four‐fold symmetry provides a platform for more complex logic systems with four potential inputs. The ring could also function as a nanoactuator for multiple proximity‐dependent reaction species attached to arm units, allowing controllable activation of the reaction [27, 28].

## Conflicts of Interest

The authors declare no conflicts of interest.

## Supporting information




**Supporting File**: smll75370‐sup‐0001‐SuppMat.pdf.

## Data Availability

The data that supports the findings of this study are available in the supplementary material of this article
