# Supplementary material for: Hoberman‐Inspired Reconfigurable DNA Origami Ring for Nanoscale Capture
Source: Small. 2026 Aug 25;22(60):e75370. doi: 10.1002/smll.75370 (PMC13711479; doi:10.1002/smll.75370)
Supplement: Supplementary file 1 [file SMLL-22-e75370-s001.pdf]

Supporting Information

Hoberman-inspired Reconfigurable DNA Origami Ring for Adaptive Nanoscale Capture

Henry Wisniewski[a], Minu Saji[a], Moisés Reyes[a], Qi Yang[a], Fei Zhang[a*]

[a]Department of Chemistry, Rutgers University, Newark, NJ 07102, USA
[*]Address correspondence to this author at the Department of Chemistry, Rutgers University, Newark, NJ 07102, USA;
Tel: 973-353-5520; E-mail: fei.zhang@rutgers.edu

Material and Methods

Preparation of the Origami Ring
The M13mp18 scaffold DNA was purchased from New England Biolabs (Catalog#: N4040S). The DNA staples were designed using caDNAano and ordered from Integrated DNA Technologies in a plate at $100\mu M$. The ring was prepared using a 1:10 ratio of M13mp18 scaffold to DNA staples at $5nM$ and $50nM$ respectively in 1xTAE buffer with $14mM$ Mg^{2+}. The mixture was annealed with the following program: 92 °C for 5 minutes, 90 °C for 5 minutes, 86 °C to 70 °C at a rate of -1 °C/15min, 69 °C to 40 °C at a rate of -1 °C/15min, 39 °C to 25 °C at a rate of -1°C/10min, then hold at 4 °C. To achieve locked conformations, the locking staples were added to the pre-annealed mixture x10 the scaffold and annealed using the same program. The design and strand information can be found in figure S1 and tables S1-S4.

Reconfiguration of the Ring
The ring was annealed using toehold-possessing locking strands following the standard protocol mentioned. Displacement strands were added at a 1:10 ratio between the locking strands and displacement strands to ensure complete displacement of the locking strands. The sample was then incubated at 50 °C for 6 hours. Afterward, the target locking and filler strands were added and annealed from 50 °C to 4 °C at a rate of -1 °C/10min for a total of 7 hours. The samples were then directly characterized.

Gold Nanoparticles Functionalization
$5nm$ gold nanoparticles were purchased from Ted Pella Inc. (Catalog #: 17600-20). Thiol-modified DNA was purchased from Integrated DNA Technologies. The gold nanoparticles, thiol-modified DNA, and SDS were added to a tube to final concentrations $60nM$, $30\mu M$, and $350\mu M$ respectively to a final volume of $300\mu L$. The sample was then shaken at 1200rpm at 30 °C covered in foil. $5\mu L$ of $2M$ NaCl was added to the sample every hour for the first 6 hours. After, the sample was shaken overnight. The sample was centrifuged at 15k rpm for 1 hour at 4 °C. The supernatant was removed from the tube and $300\mu L$ of ultrapure water was added. The sample was vortexed and centrifuged again. This was repeated 3 times. A final addition of $30\mu L$ of ultrapure water was added and the functionalized nanoparticles were stored in the dark at 4 °C.

Gold Nanoparticle Attachment
DNA attachment probes were purchased from Integrated DNA Technologies as dry tubes. The attachment probes were annealed with the ring at a 1:2 ratio between M13mp18 and the probes. Once annealed, the functionalized gold nanoparticles were added at a 1:1 ratio with the attachment probes. The mixture was then annealed with the following program: 45 °C to 5 °C with a rate of -1 °C/18min, then held at 4 °C. The sample was then directly characterized.

Preparation of Nanostructures
All DNA strands for the nanostructures were ordered through Integrated DNA Technologies and hydrated to $100\mu M$. The strands were then used as is, without purification. The DNA strands were mixed in 1xTAE

Mg^{2+} buffer (12.5mM Mg^{2+} for the xTile and Needle tile; 20mM Mg^{2+} for the tetrahedron) at a 1:1 ratio between all strands. The annealing mixtures were then annealed in the following program:

Tetrahedron: 95 °C → 75 °C → 60 °C → 45 °C → 30 °C → 20 °C each temperature held for 2 minutes, then held at 4°C.

Needle Tile and X tile: 94 °C - 86 °C at -4 °C/5 minutes, 85 °C - 70 °C at -1°C/5min, 69 °C - 40 °C at -1 °C/15 min, 39 °C - 25 °C at -1°C/10min, and held at 4 °C.

All nanostructures were then directly used without purification.

Capture of Nanostructures on Origami Ring
The origami and nanostructures were first annealed individually. Then the glue strands were added to the nanostructures at a 1:1 ratio between glue strand and anchor probes (3 glue strands/nanostructure). A Cy5-modified DNA was added at a 1:1 ratio per nanostructure. The sample was then incubated for 1 hour at 37 °C. The funtionalized nanostructures and origami were mixed at a 1:1 ratio and annealed from 37 °C to 15 °C at a rate of -1 °C/30min then held at 4 °C.

Gel Electrophoresis
The DNA origami was characterized using a 0.7% agarose gel in 1xTAE (12.5mM Mg^{2+}) buffer. 10μL of sample with 1μL of tracking dye was added to each well. The gel was run at 80V for approximately 3 hours in a water bath at room temperature. The gel was stained with GelRed (Supplier No.41003) from VWR and imaged using the Biorad Gel Doc XR Imaging System. The nanostructure attached origami was imaged using a BioRad ChemiDoc MP Imaging System, with all exposures set to optimal auto-exposure settings, looking at Cy5 and GelRed emissions.

Atomic Force Microscopy
The DNA origami were imaged using Bruker's Dimension FastScan AFM in ScanAsyst in Liquid mode. The ScanAsyst-Fluid+ tip from Bruker was used. 5μL of 5nM origami was deposited on a freshly cleaved mica surface followed by 10μL of 1xTAE (12.5mM Mg^{2+}) and left undisturbed for 3 minutes. The mica surface was then washed 4 times with 70μL of the buffer (ultrapure water for gold nanoparticle attached origami). 2μL of 100mM NiCl and a final 70μL of buffer was added before imaging.

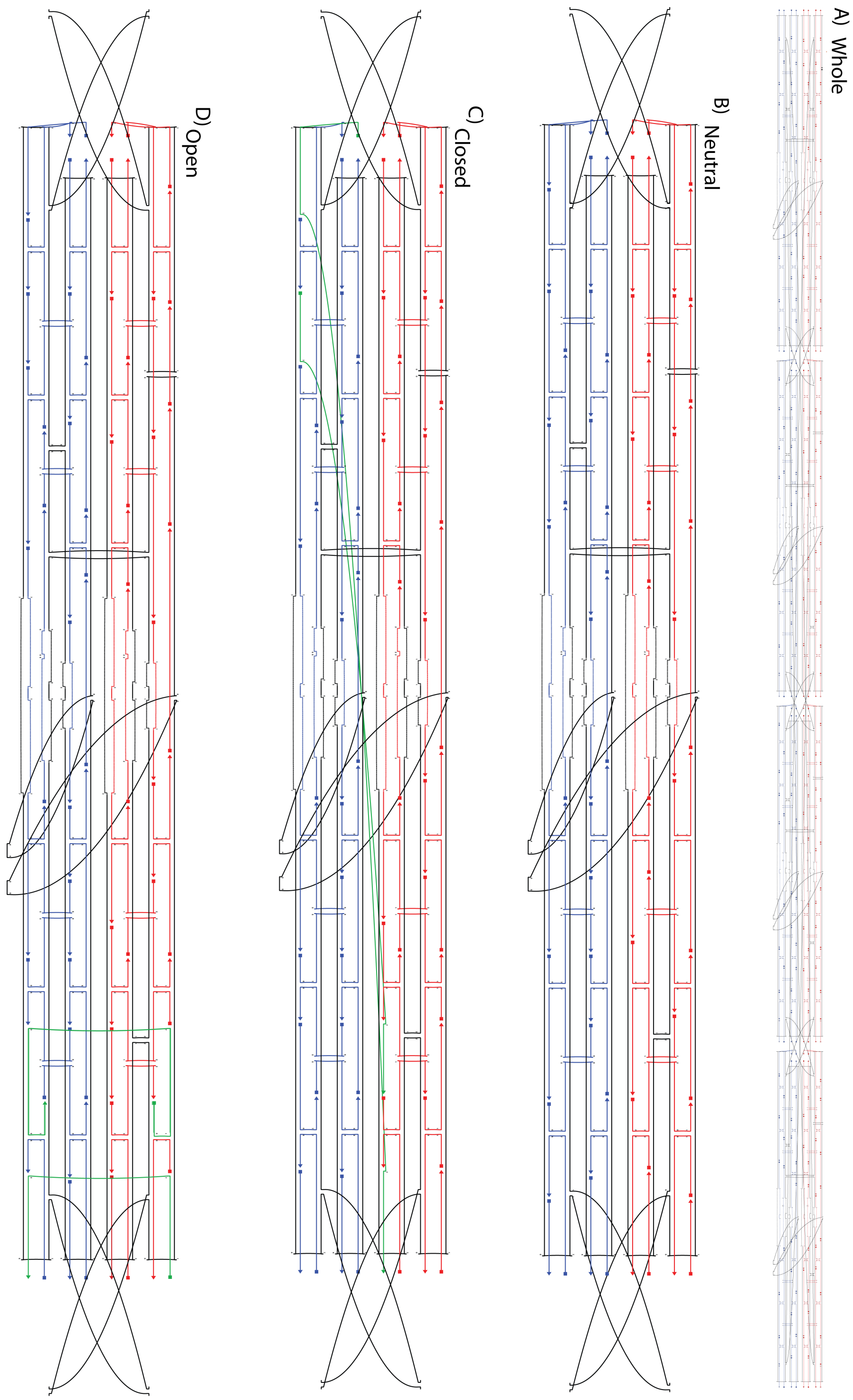

Figure S1: CaDNAno design of DNA origami ring. (a) Entire caDNAno design of ring. Red = top layer, blue = bottom layer, black = scaffold. (b) Zoomed caDNAno of a scissor unit for the neutral conformation. (c) Zoomed caDNAno of a scissor unit for the closed conformation. Green = locking strands. (d) Zoomed caDNAno of a scissor unit for the open conformation. Green = locking strands.

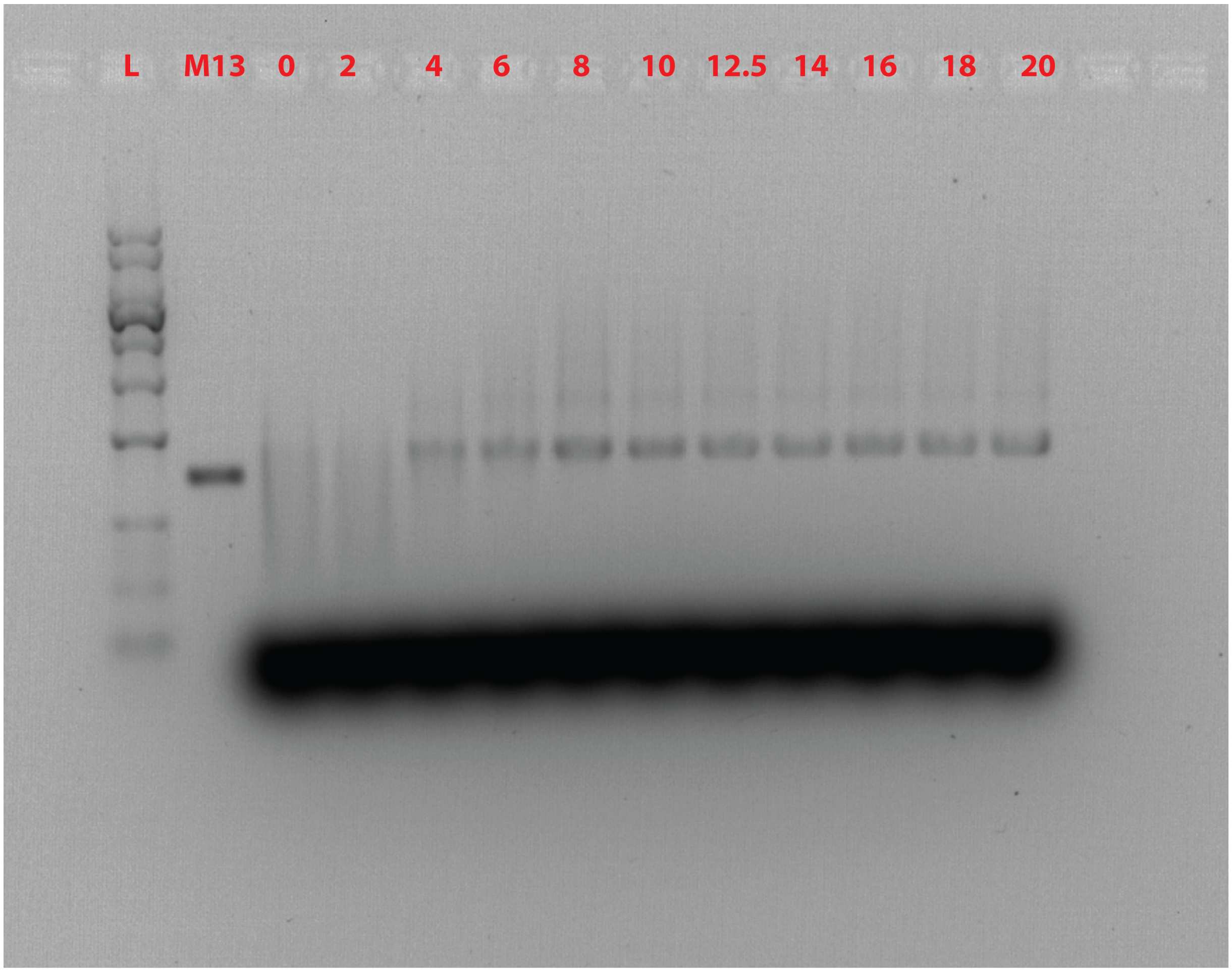

Figure S2: Magnesium buffer titration study. L: 1kb DNA ladder, M13: Un-annealed M13mp18 scaffold DNA, 0-20: mM Mg^{2+} in TAE buffer used for annealing DNA origami.

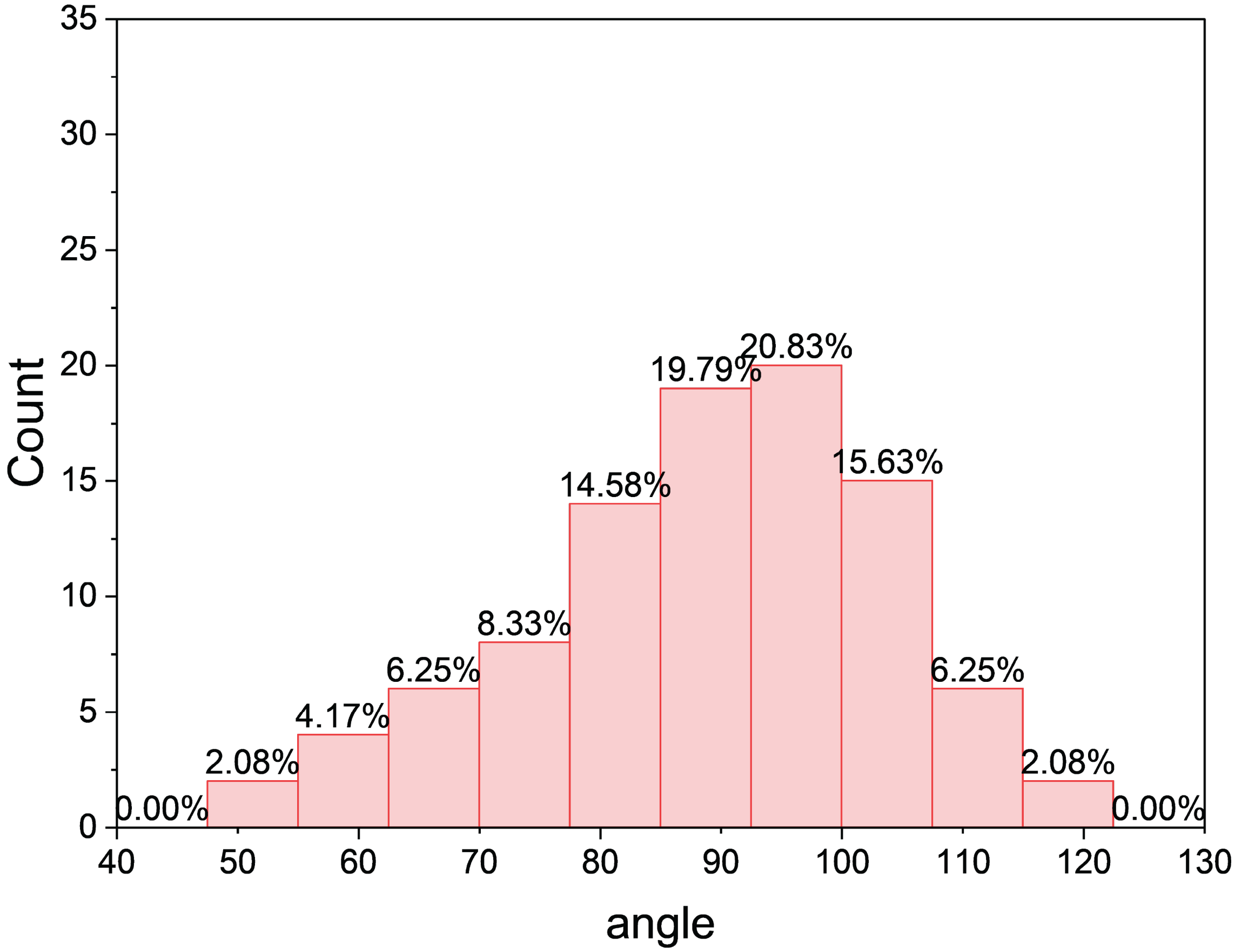

Figure S3: Angle measurements of the ring using ImageJ plotted as a histogram. Average arm angle = 89.07 degrees (SD = 14.96, N = 96).

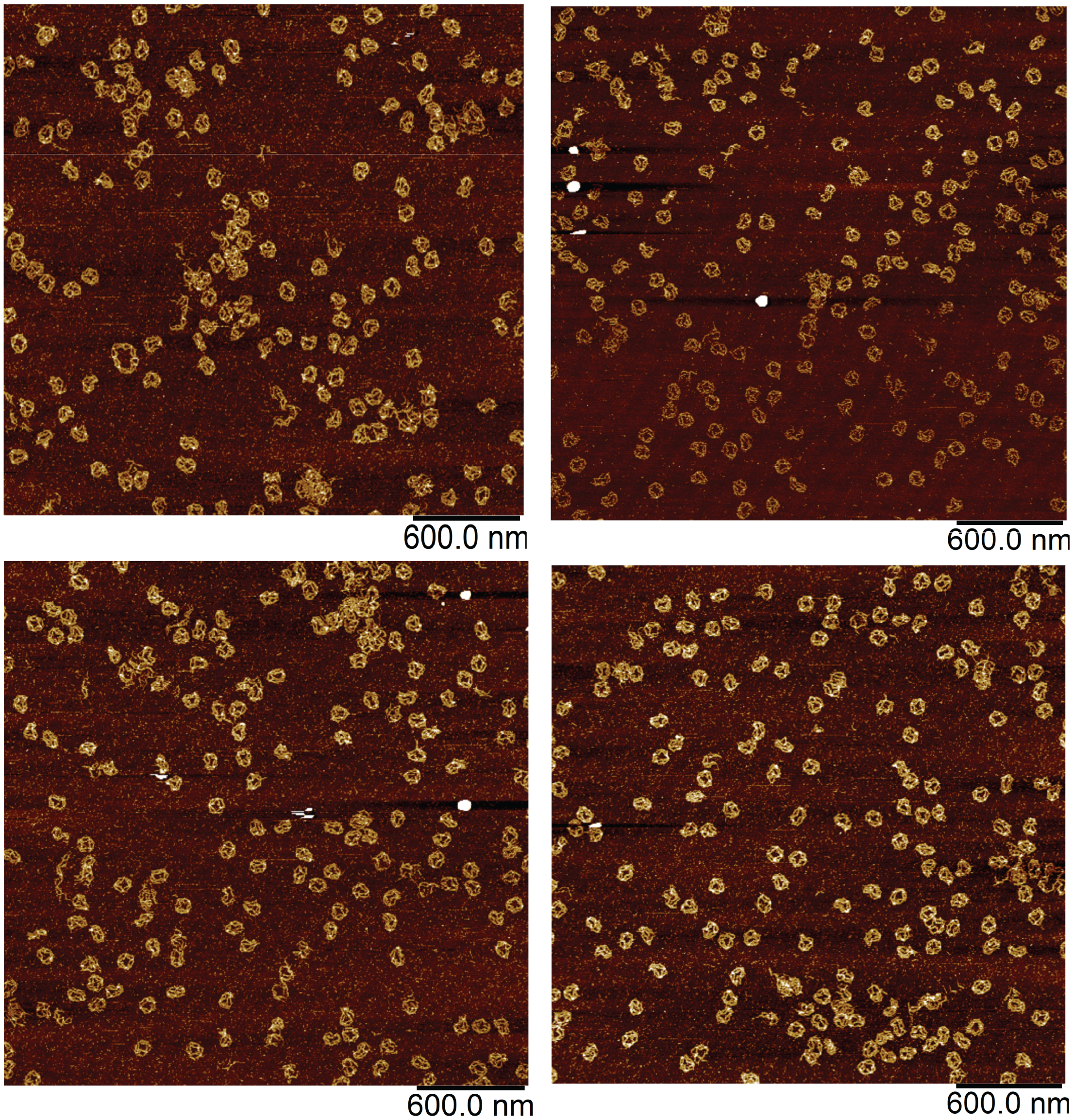

Figure S4: AFM of neutral DNA origami ring.

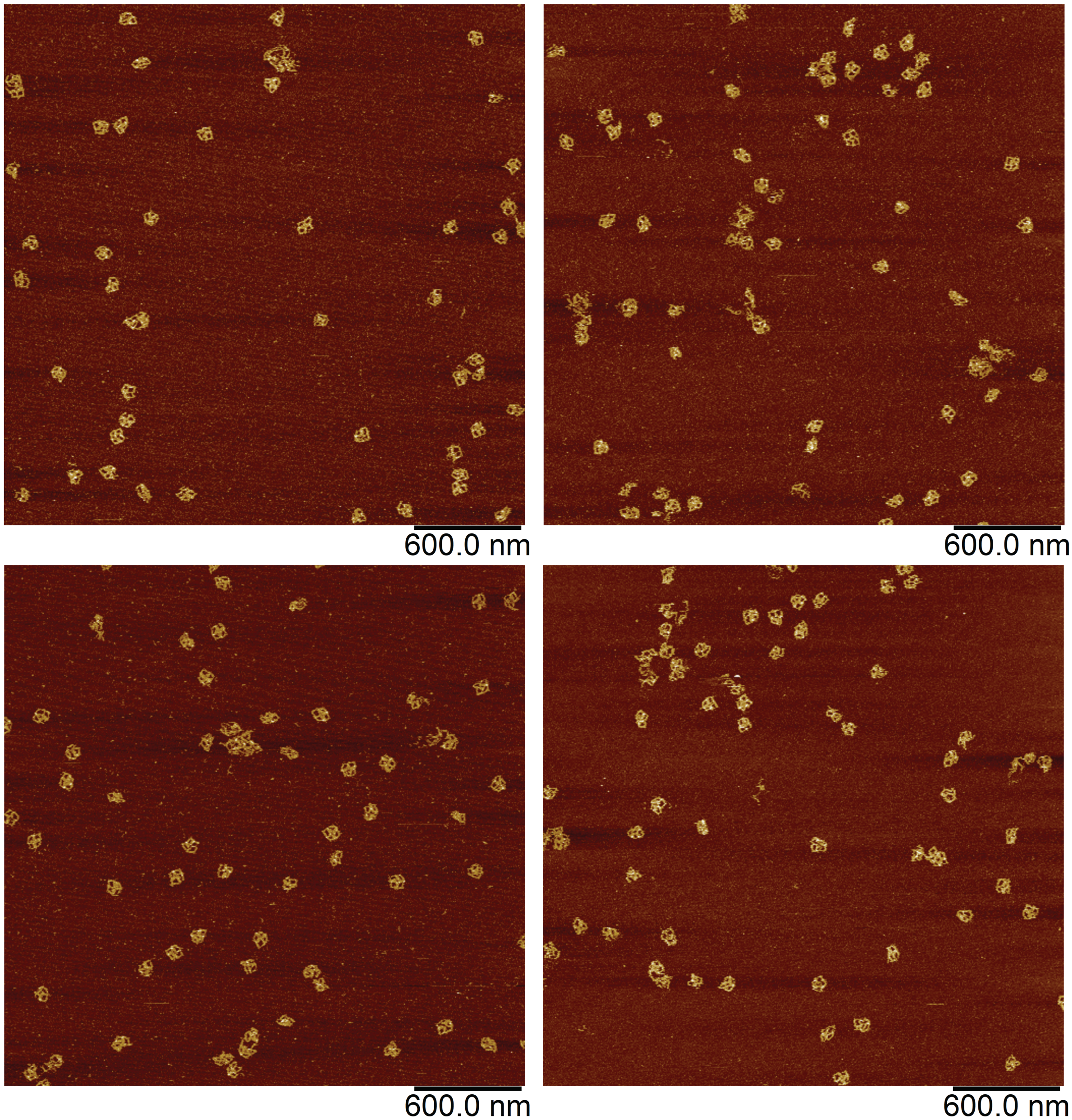

Figure S5: AFM of closed-locked DNA origami ring.

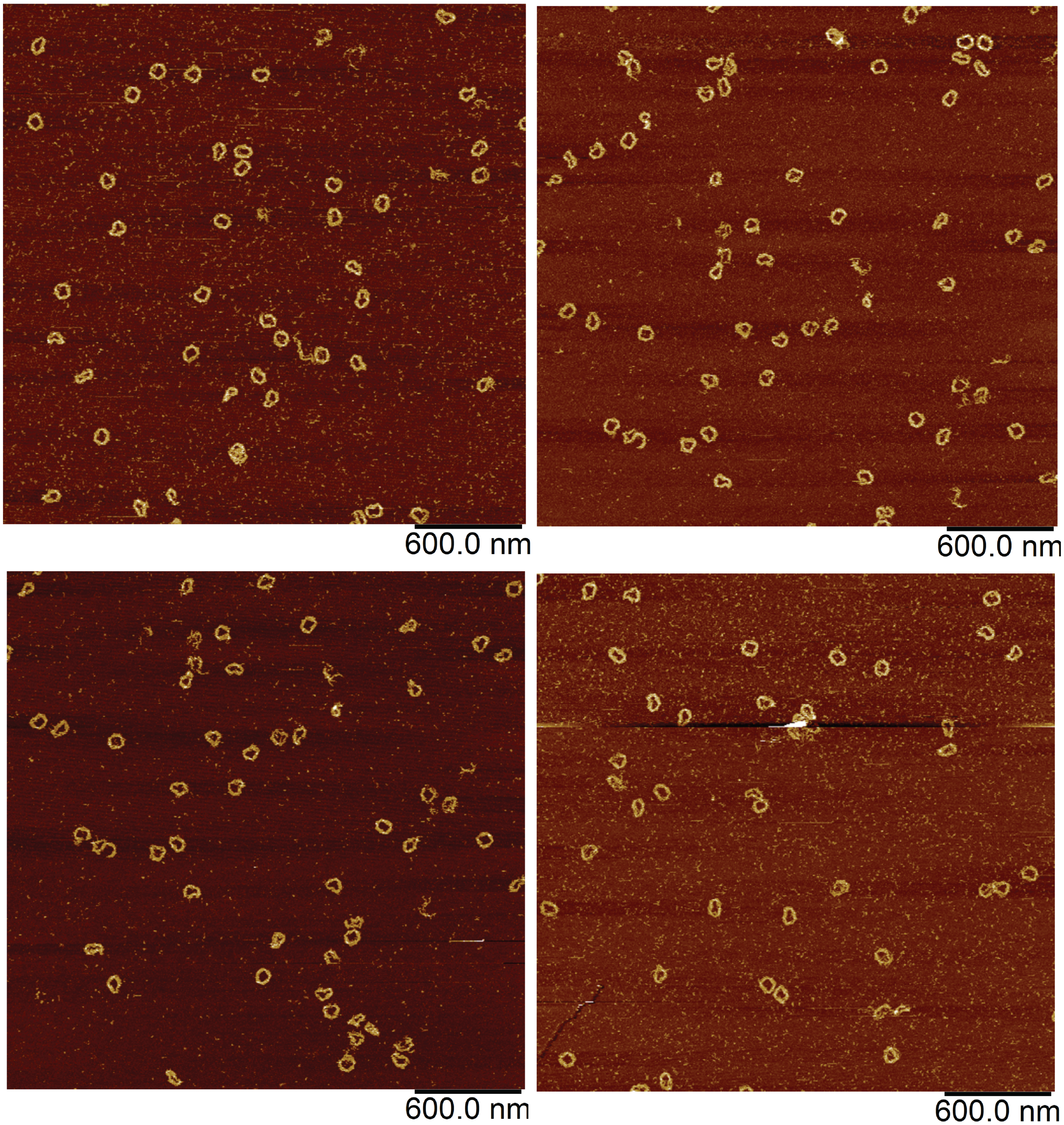

Figure S6: AFM of open-locked DNA origami ring.

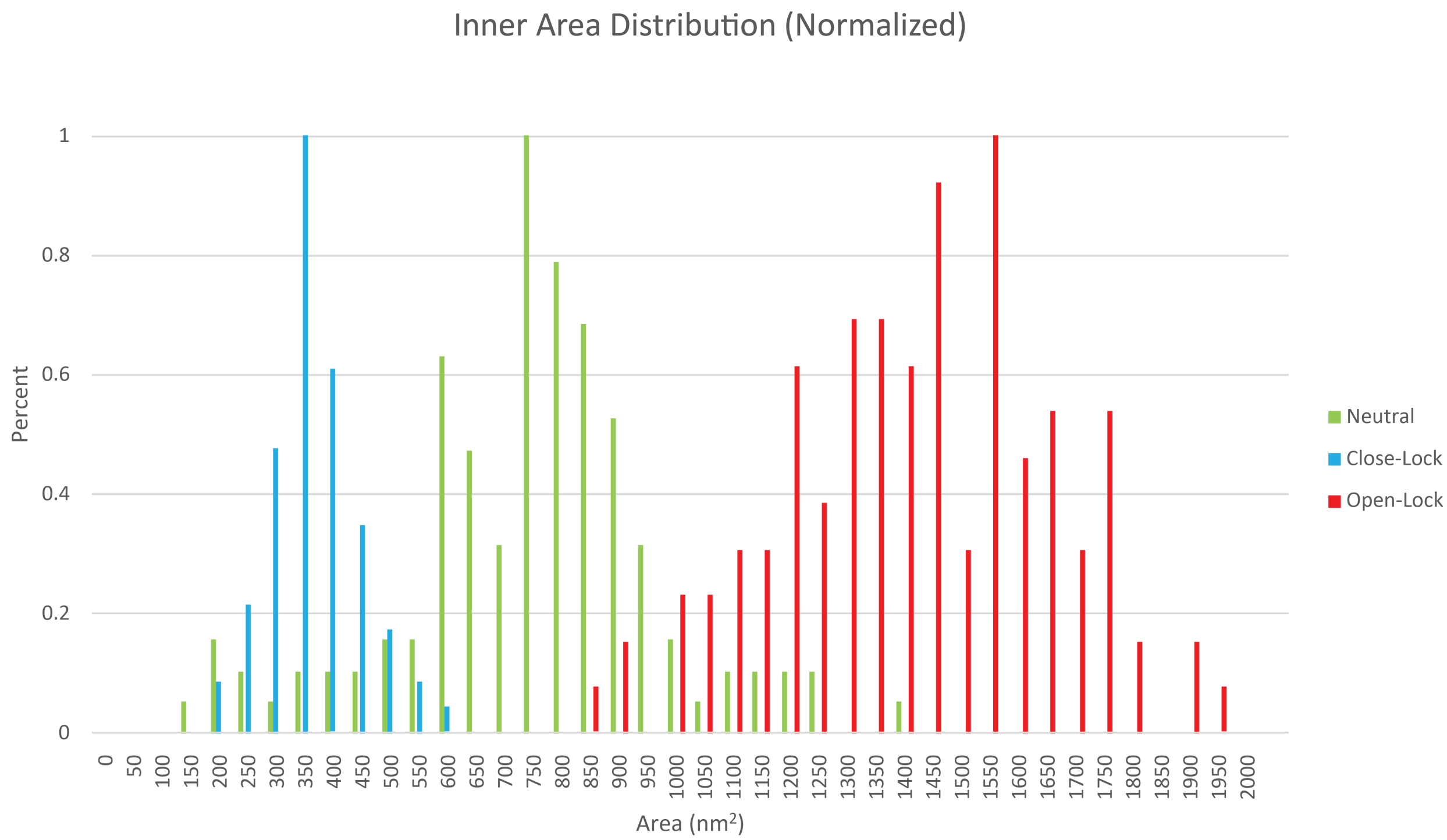

Figure S7: Normalized histogram of the calculated inner areas for the closed (blue), neutral (green), and open (red) locked conformations of the DNA ring (Closed: N = 70, neutral: N = 122, and open: N = 114).

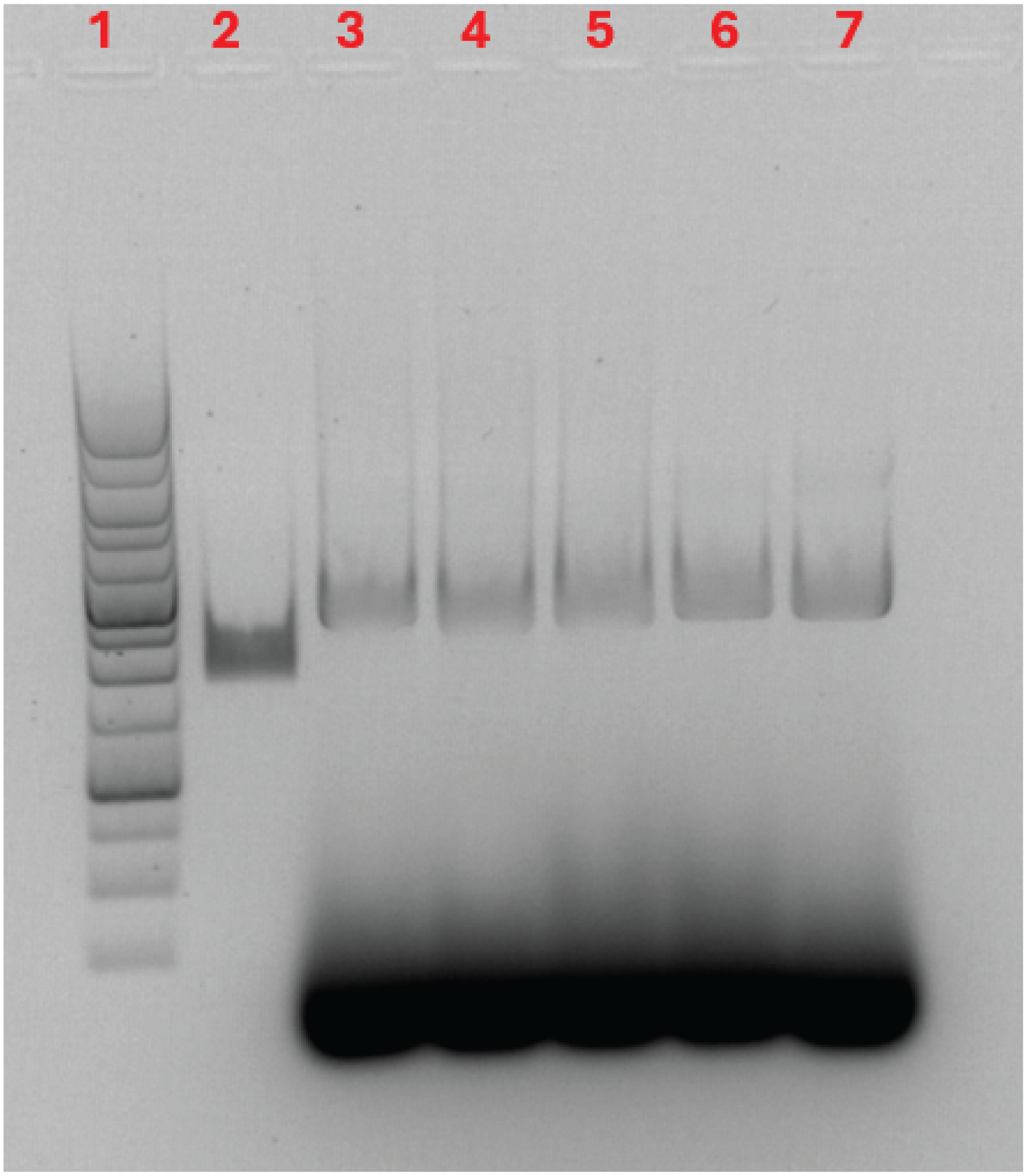

Figure S8: Buffer study for ring conformations. Lane 1: 1kb DNA Ladder, lane 2: Unannealed M13mp18, lanes 3-5: Close, neutral, and open conformations in 1xTAE 14mM Mg^{2+}, lane 6: Neutral conformation in 100mM KCl, 40mM HEPES, 14mM Mg^{2+}, and lane 7: Neutral conformation in 100mM KCl, 40mM HEPES, 1mM Mg^{2+}

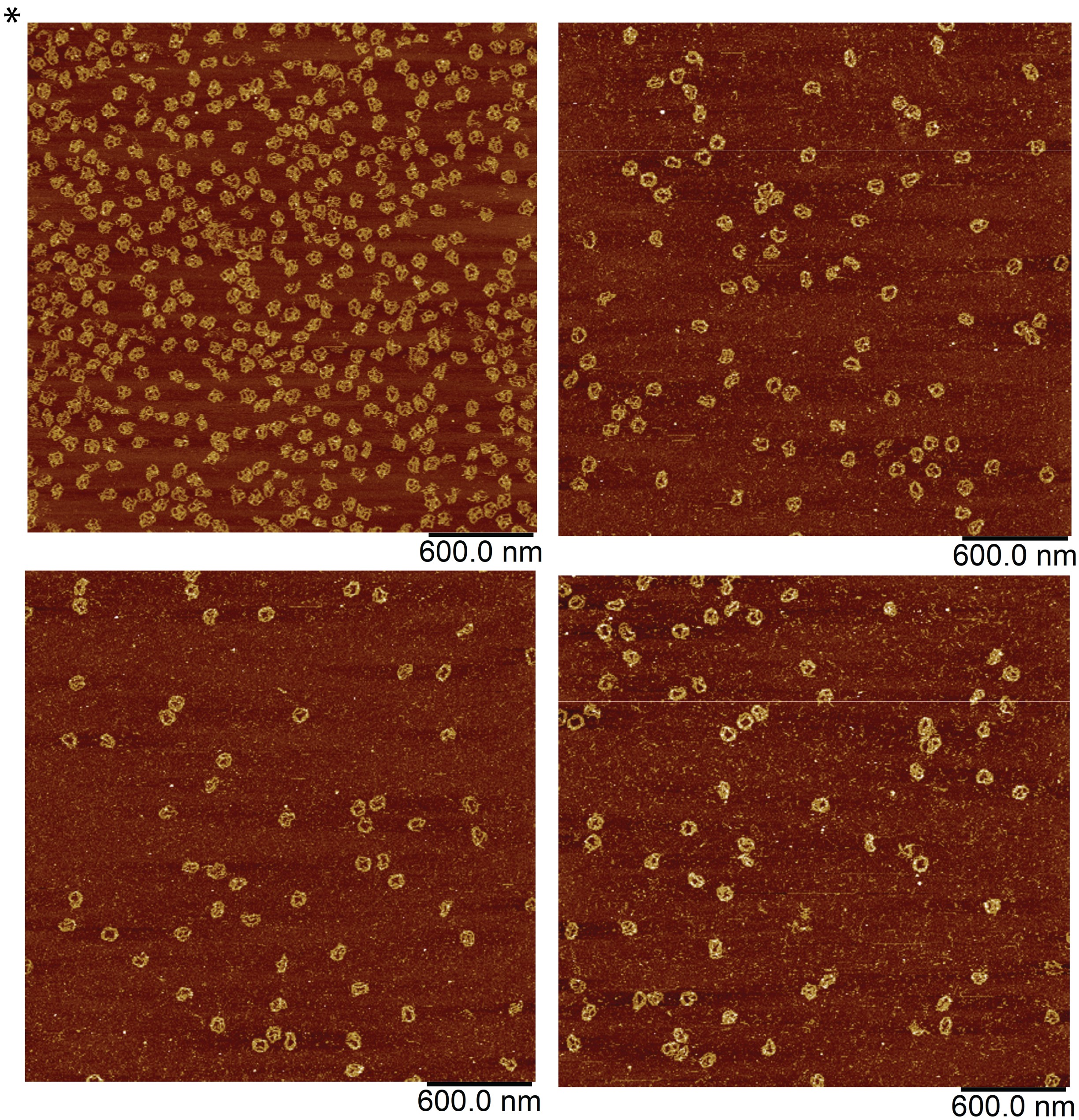

Figure S9: Closed to open reconfiguration of the ring. Top left (*) shows closed ring before reconfiguration. Other AFM are after reconfiguration.

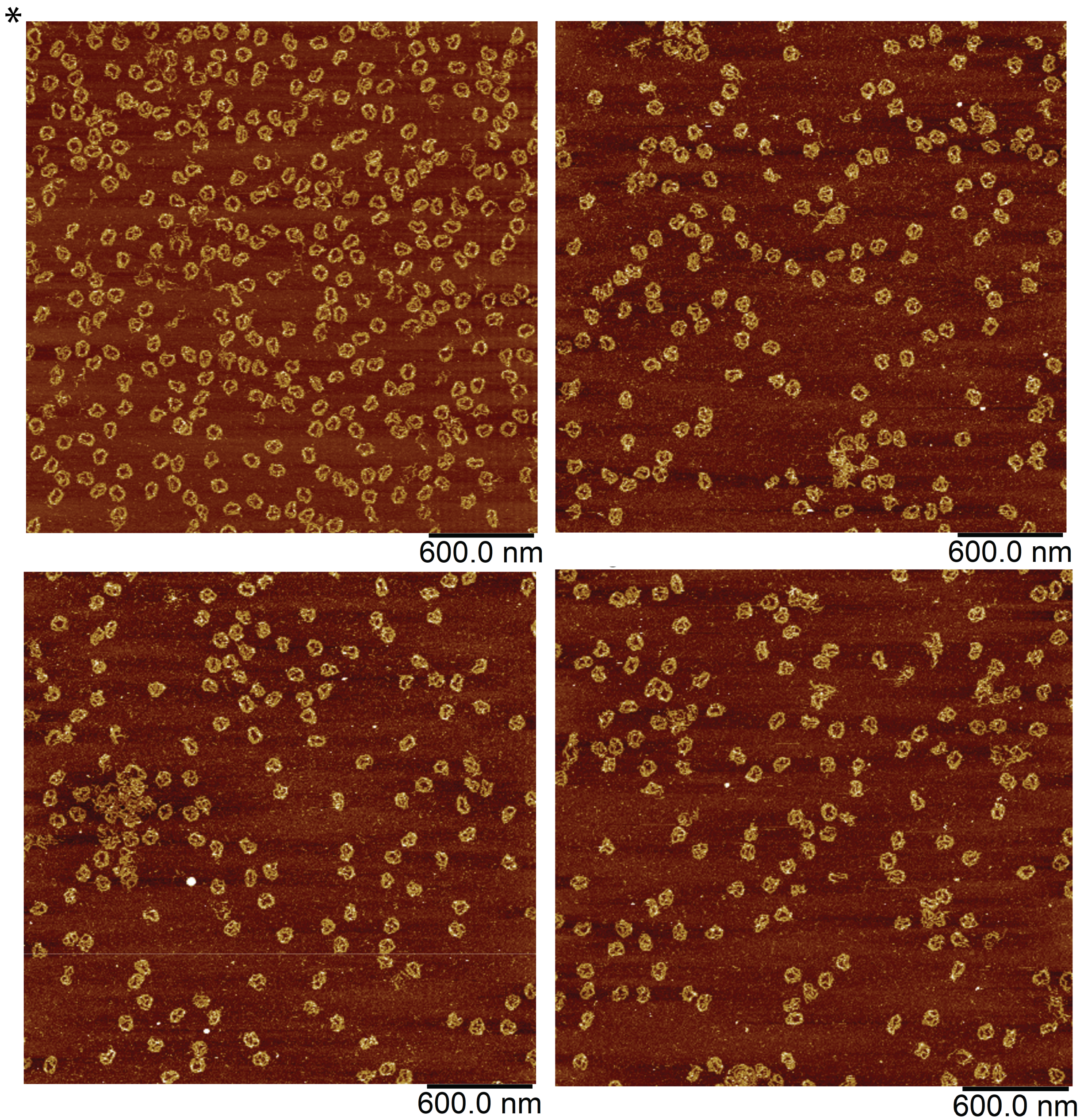

Figure S10: Open to closed reconfiguration of the ring. Top left (*) shows open ring before reconfiguration. Other AFM are after reconfiguration.

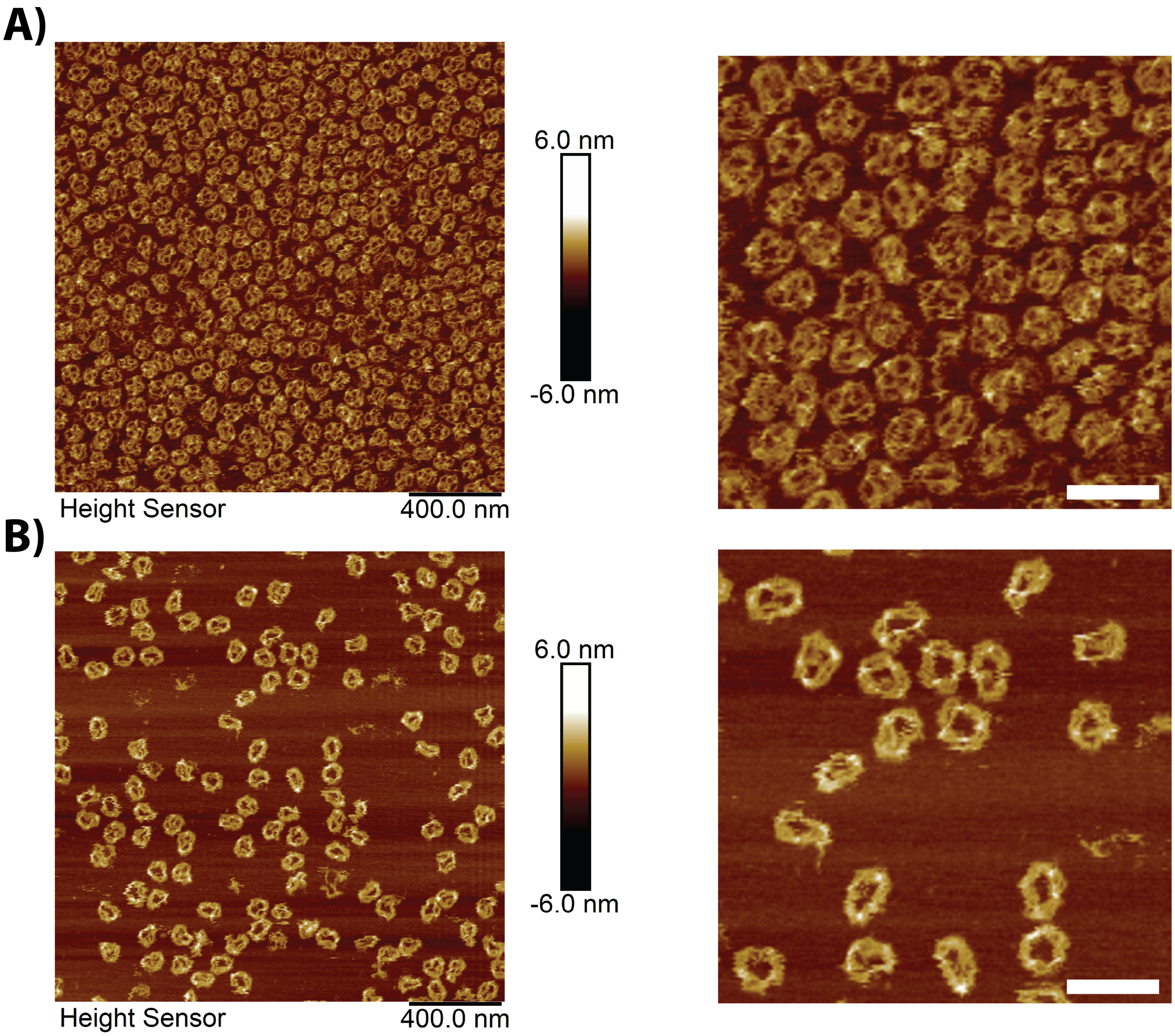

Figure S11: AFM of the ring with 2-pairs of locking strands added at opposite sides to achieve A) closed conformation and B) closed conformation. Zoomed-in scaled bar = 200 nm.

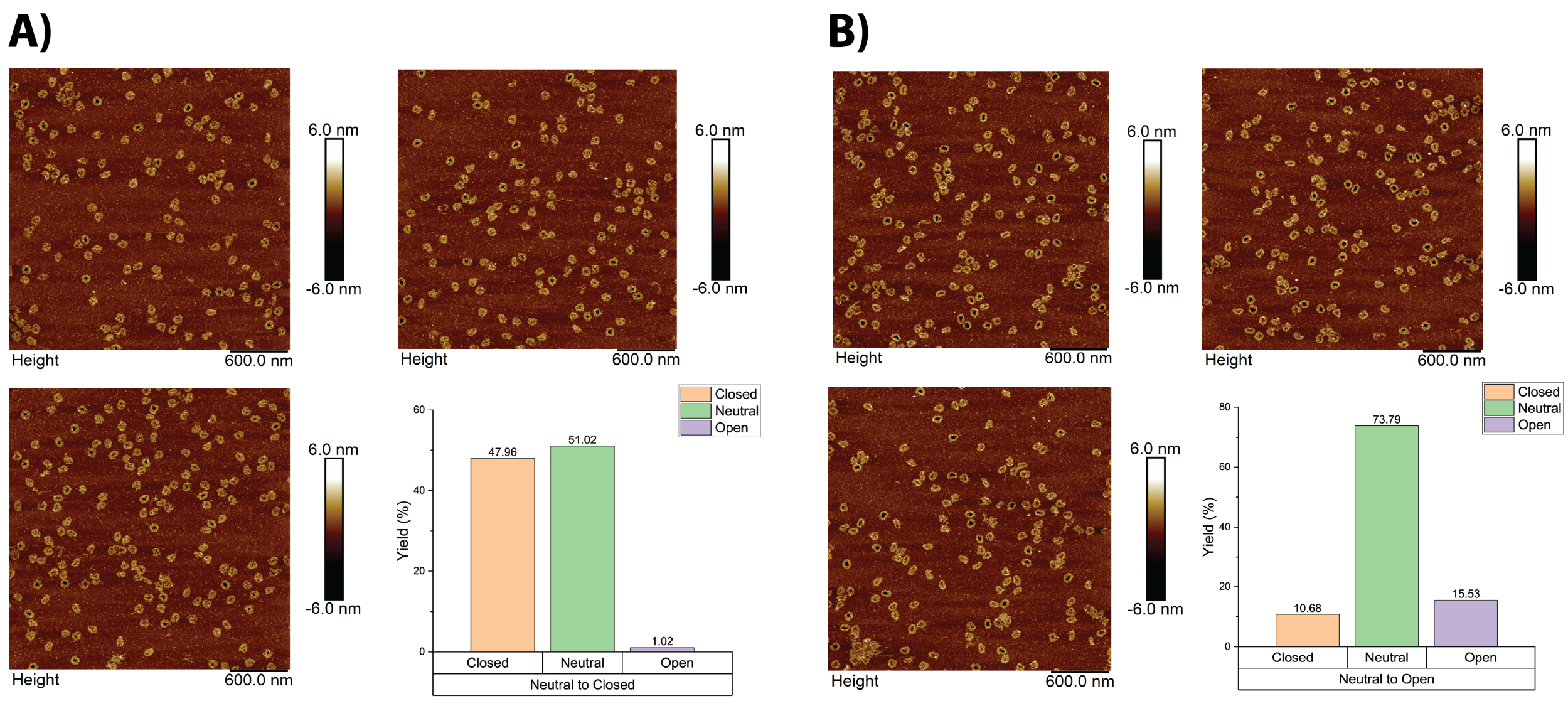

Figure S12: Reconfiguration of the ring from the neutral state to the A) closed and B) open states. Inner areas were found after the reconfiguration for each and percentages were plotted. (Neutral to closed N = 98, Neutral to open N = 103).

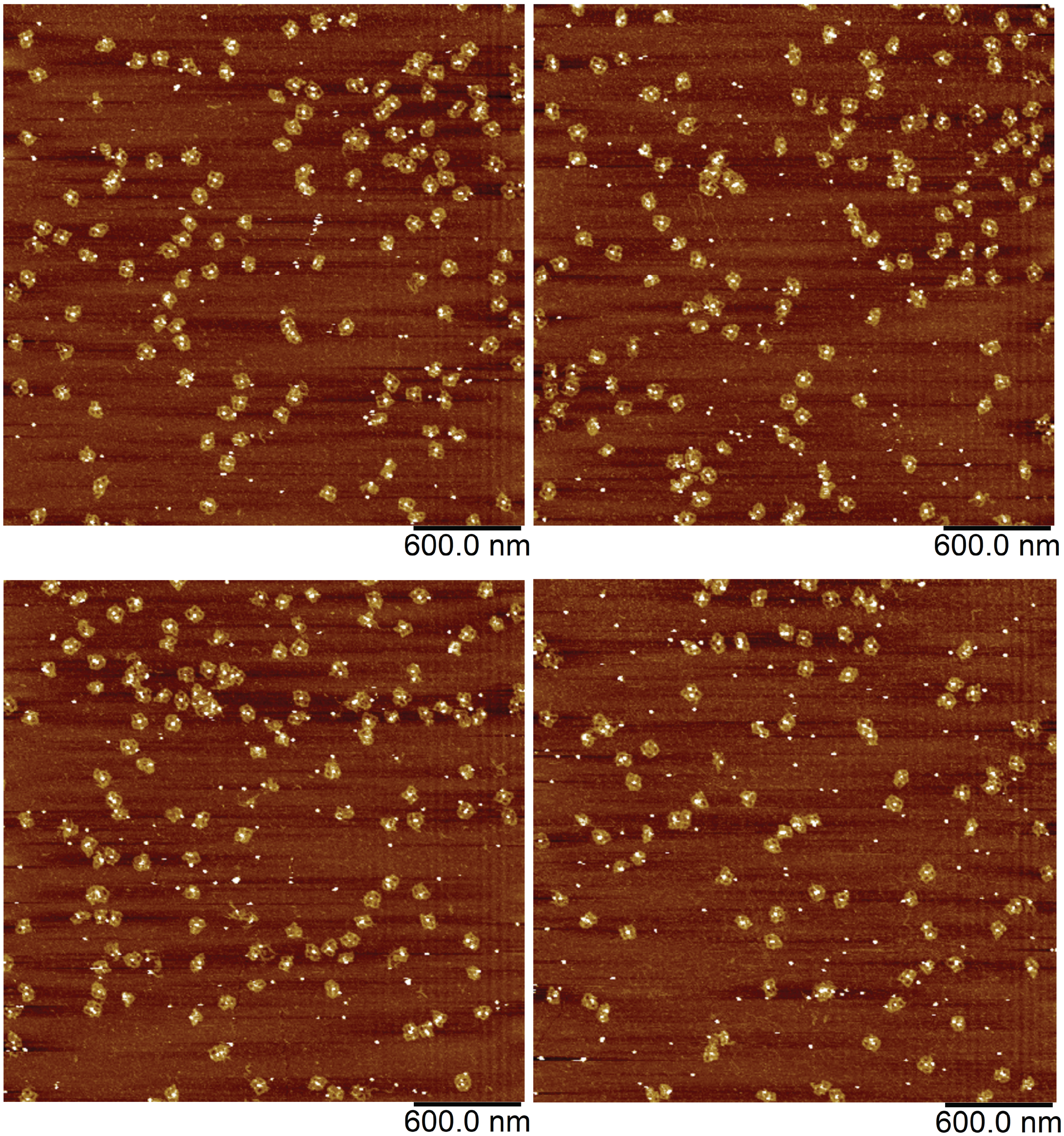

Figure S13: AFM of the closed ring binding with gold nanoparticles.

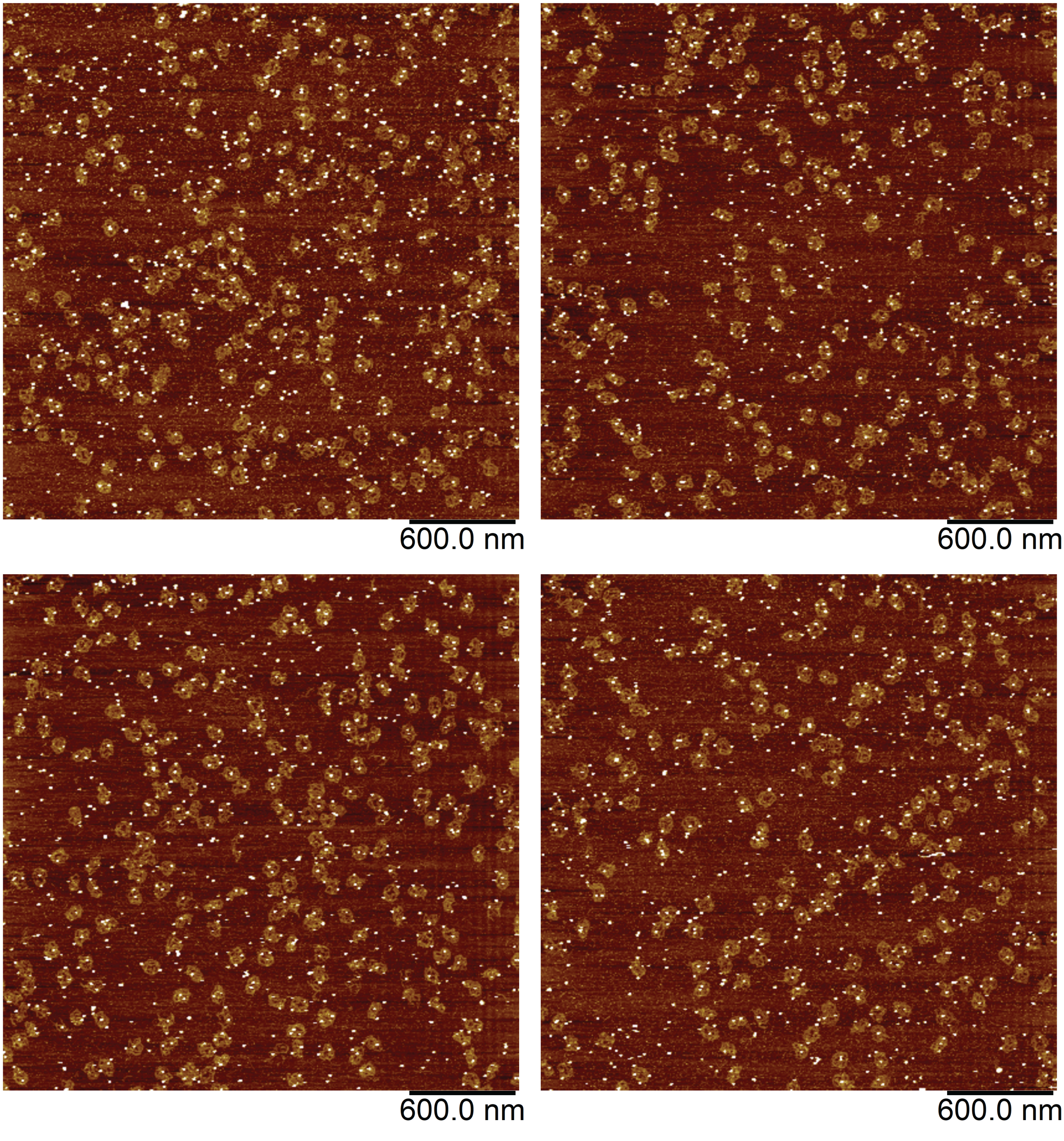

Figure S14: AFM of the neutral ring binding with gold nanoparticles.

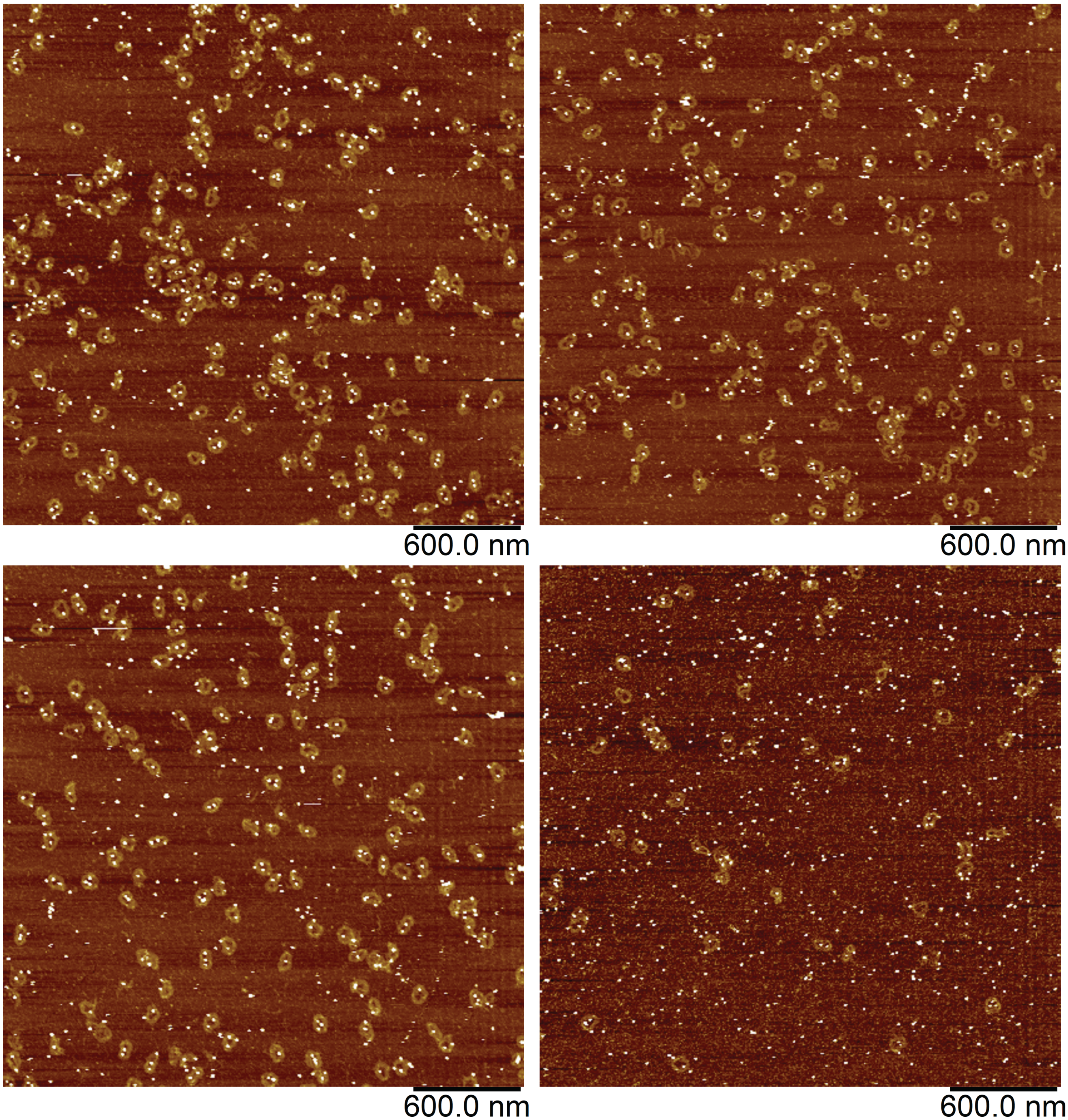

Figure S15: AFM of the open ring binding with gold nanoparticles.

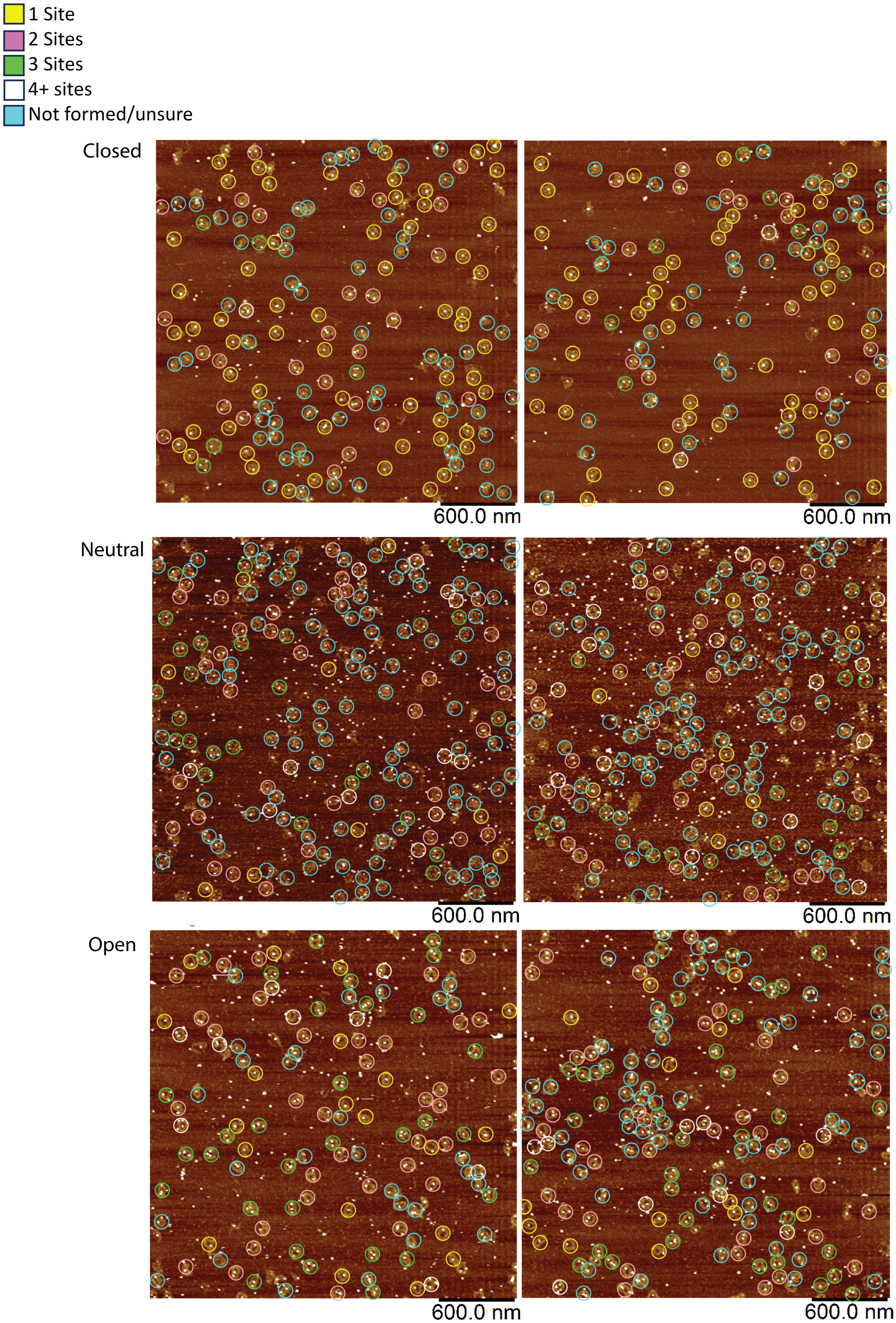

Figure S16: Gold nanoparticle attachment site yield counting (N = 170, 155, and 190 for the closed, neutral, and open conformations respectively).

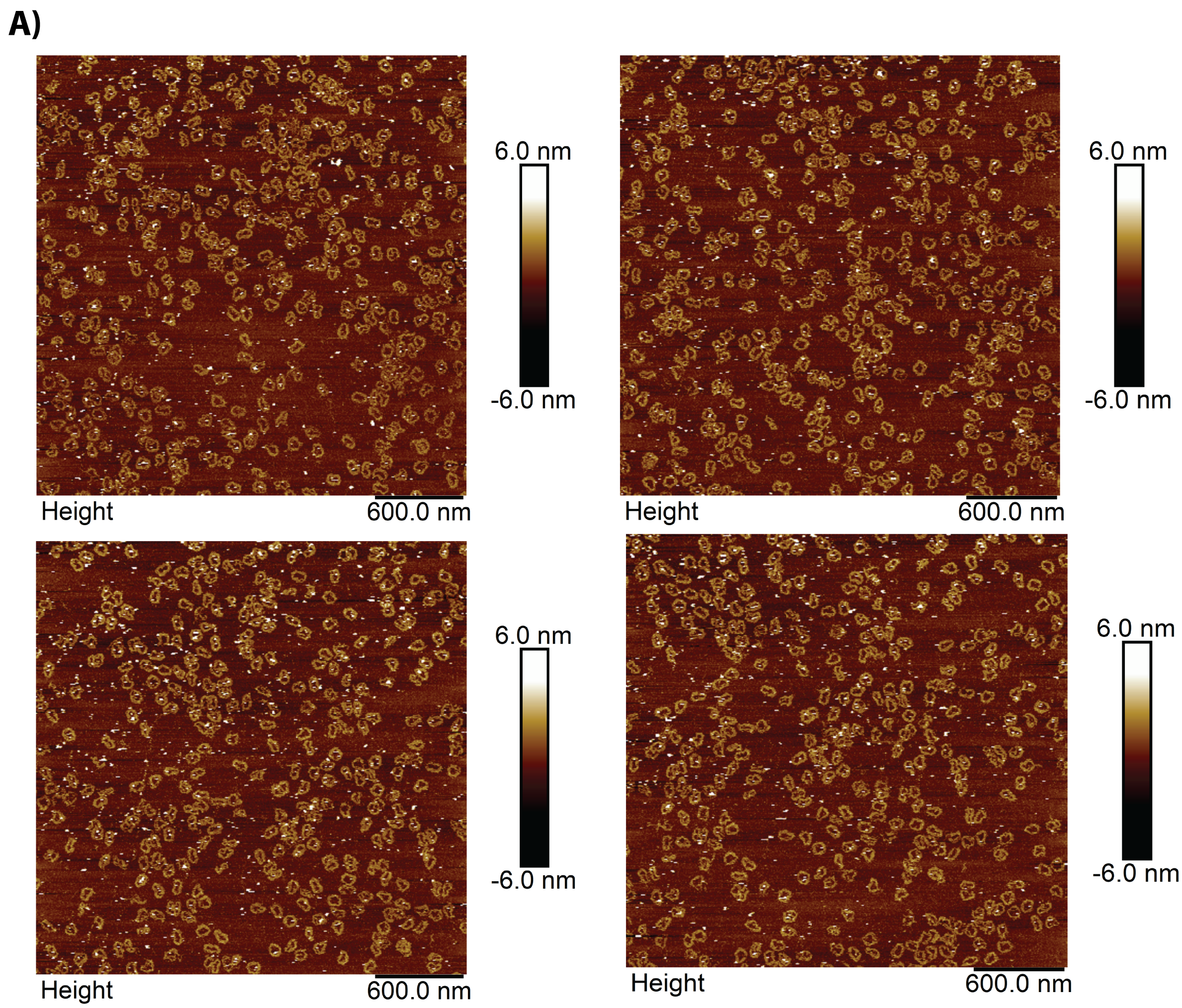

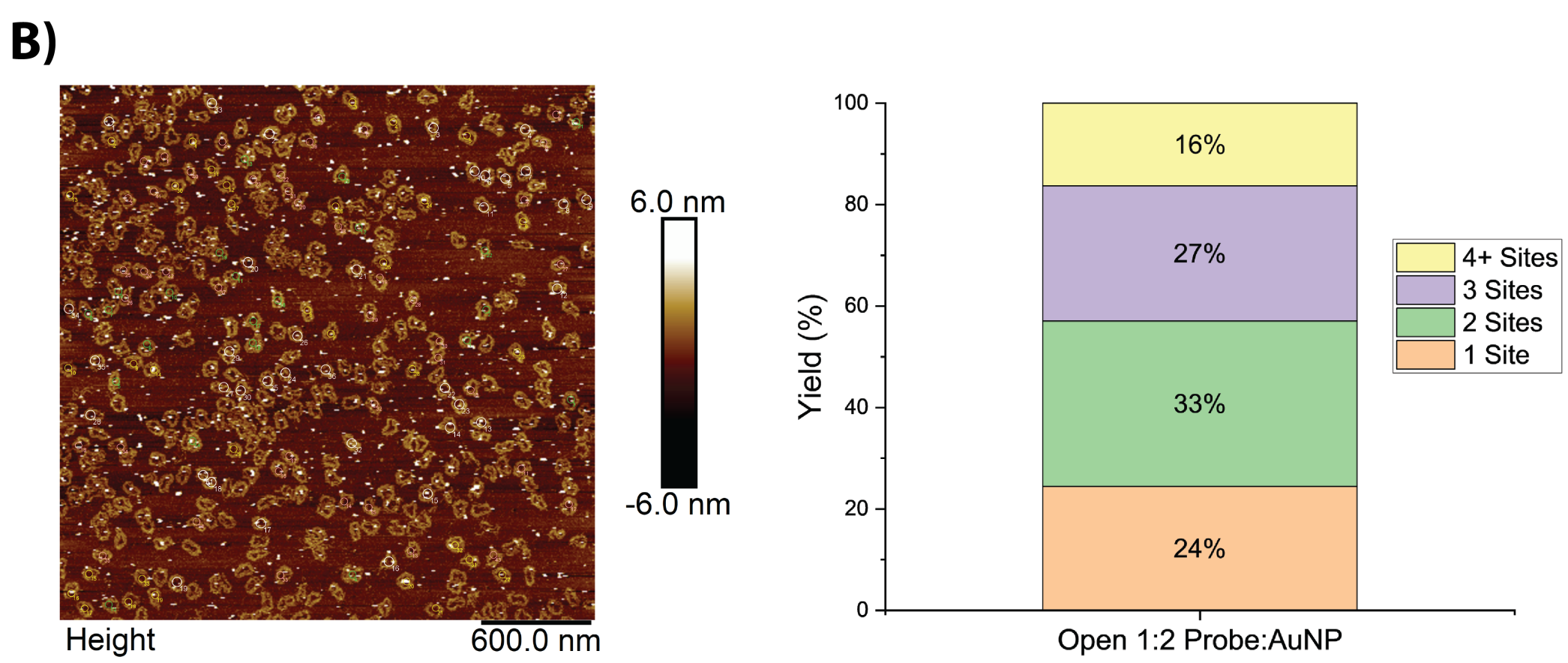

Figure S17: Gold nanoparticle capturing onto the open ring at a 1:2 ratio between the captue probes and nanoparticles. A) AFM of the gold nanoparticle attachment. B) Gold site occupancy analysis looking at total number of attachment sites per ring (N = 135). The associated plot is given on the right.

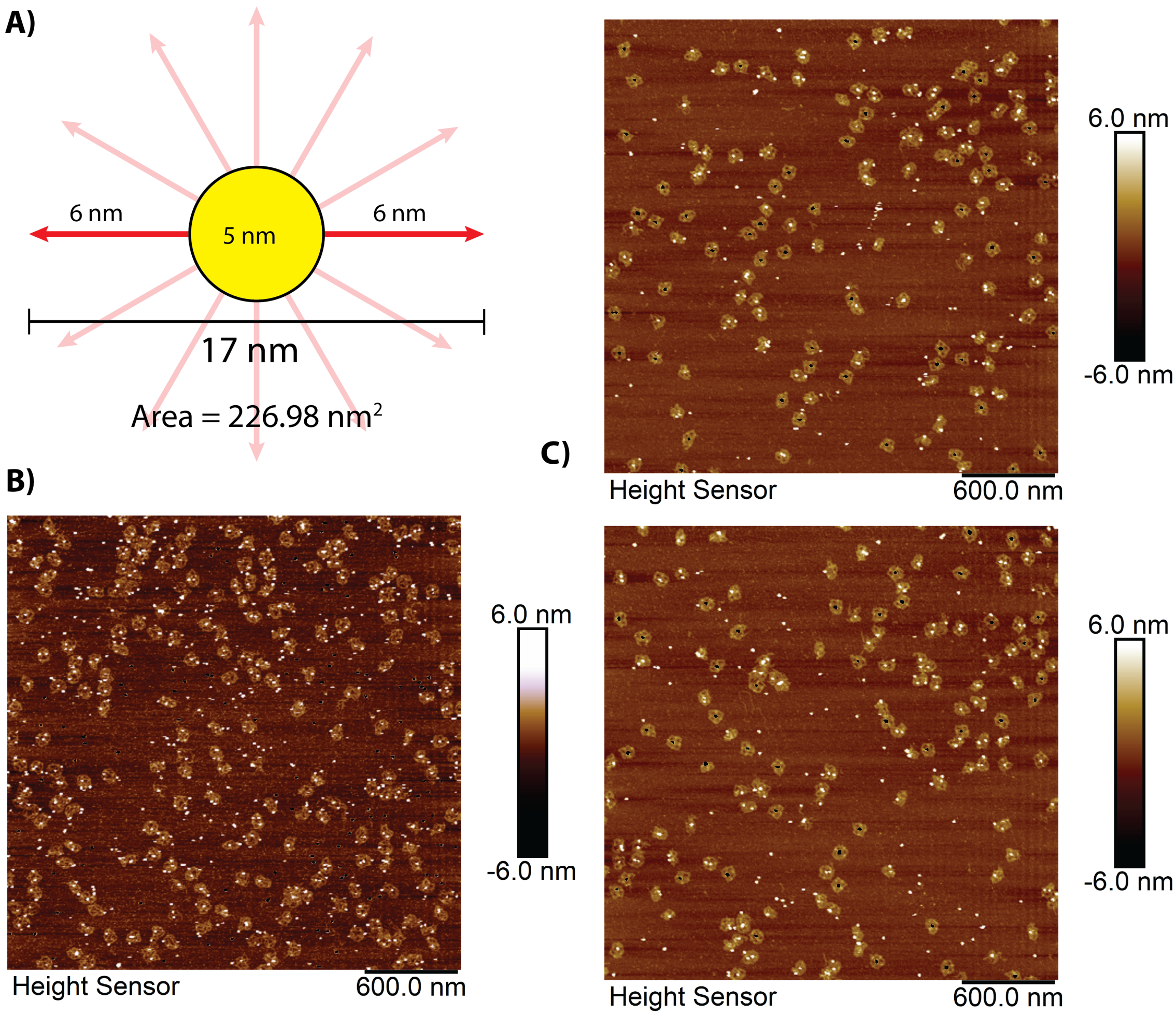

Figure S18: Gold nanoparticle analysis. A) Diagram of functionalized AuNP with theoretical diameter and area given. B) AFM analysis of free AuNP. (N=100). C) AFM analysis of AuNP at the center of the closed ring (N = 94).

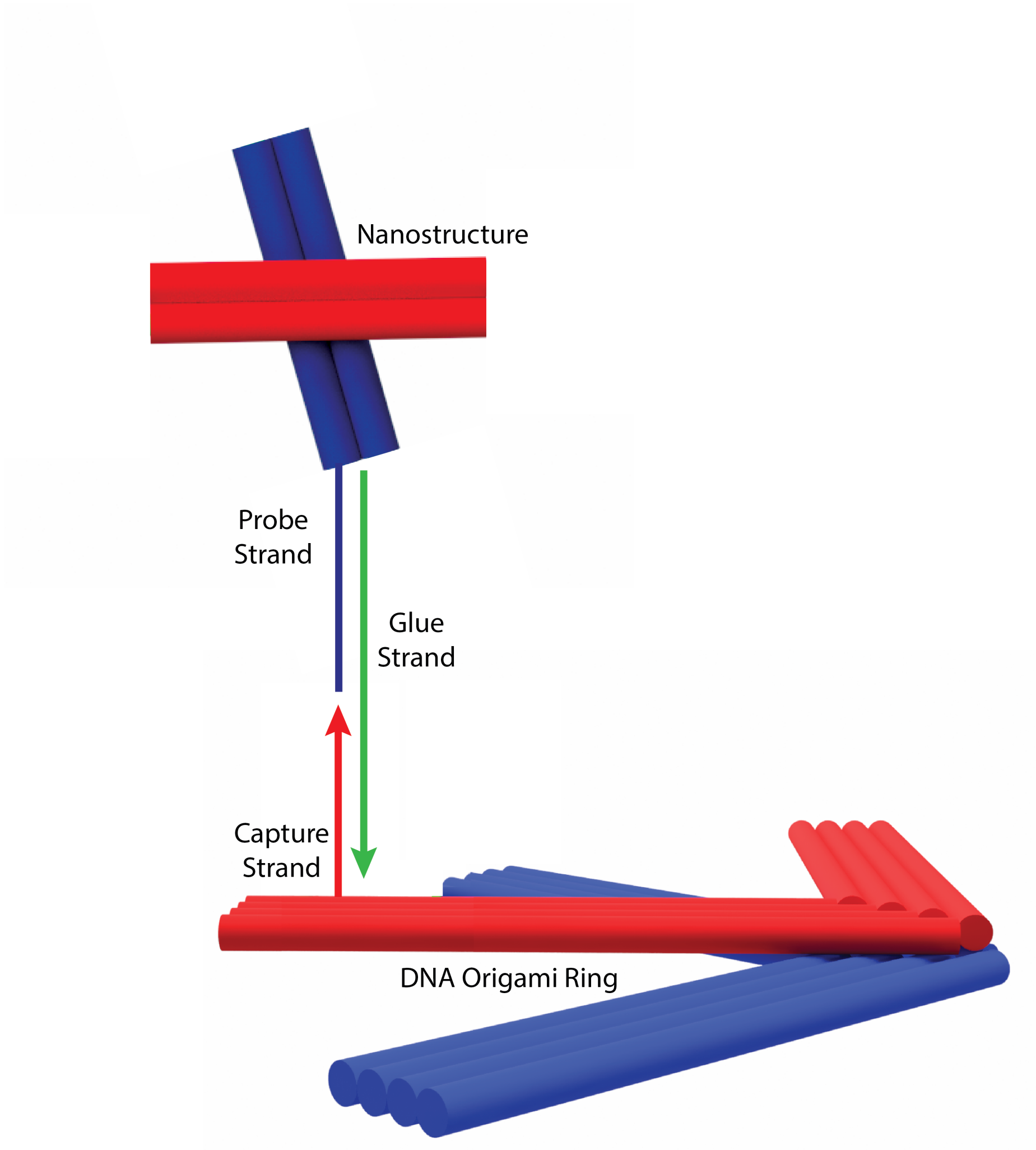

Figure S19: Schematic of the attachment of a DNA nanostructure to the DNA origami ring. Capture strand protrudes from DNA origami, anchor strand protrudes from nanostructure, and glue strand is added individually.

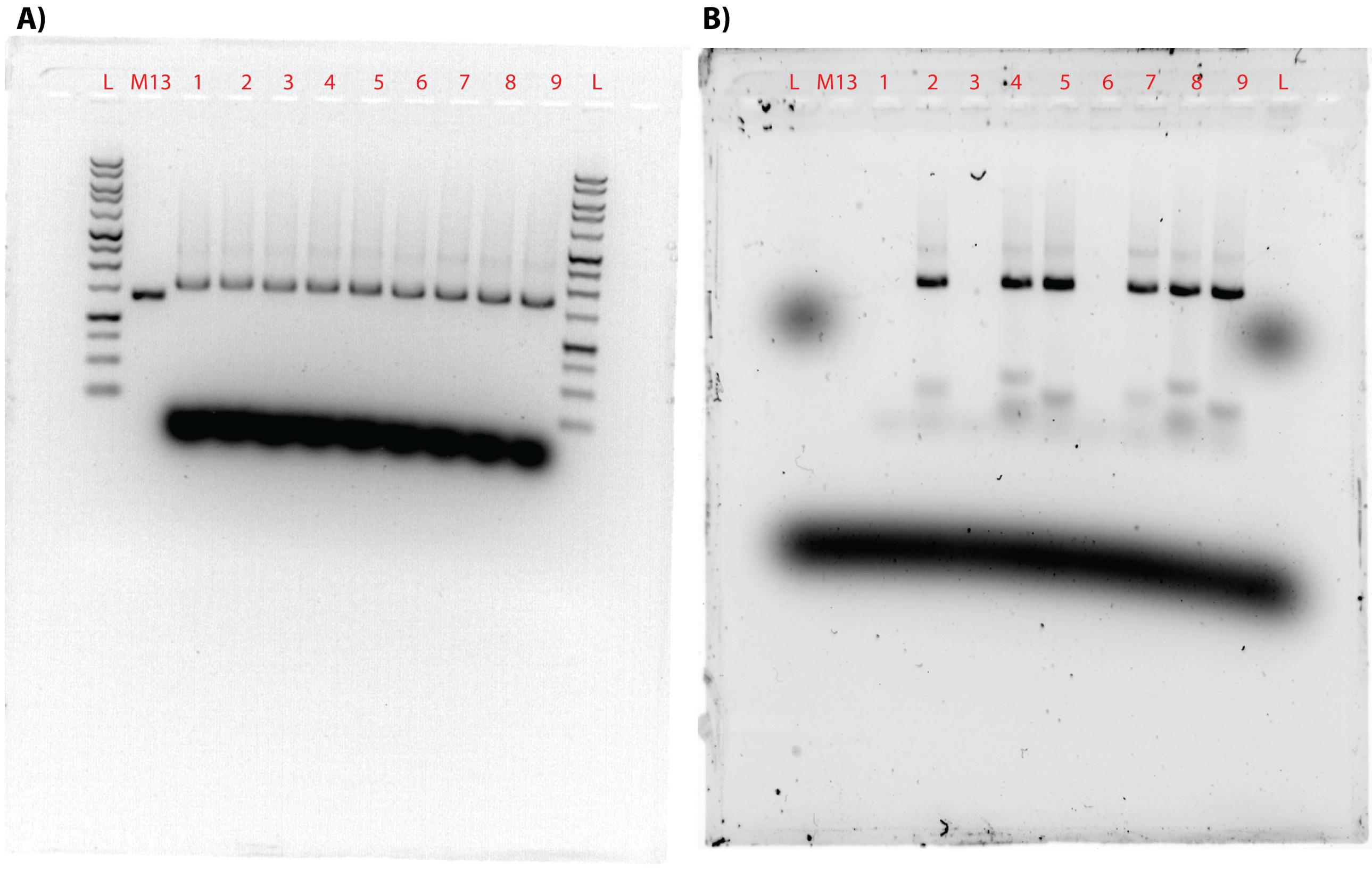

Figure S20: Nanostructure-DNA origami capturing. A) Imaged looking at GelRed emission signal. B) Imaged looking at Cy5 emission signal. The following are the gel lane descriptions, which are the same across both gels. L: 1kb DNA ladder, M13: Un-annealed M13mp18 scaffold DNA, lane 1: Closed ring, lane 2: Closed ring with tetrahedron nanostructure, lane 3: Open ring, lanes 4-5: Open ring with xTile and Needle nanostructure respectively, lane 6: Neutral ring, lanes 7-9: Neutral ring with tetrahedron, xTile, and Needle nanostructures respectively.

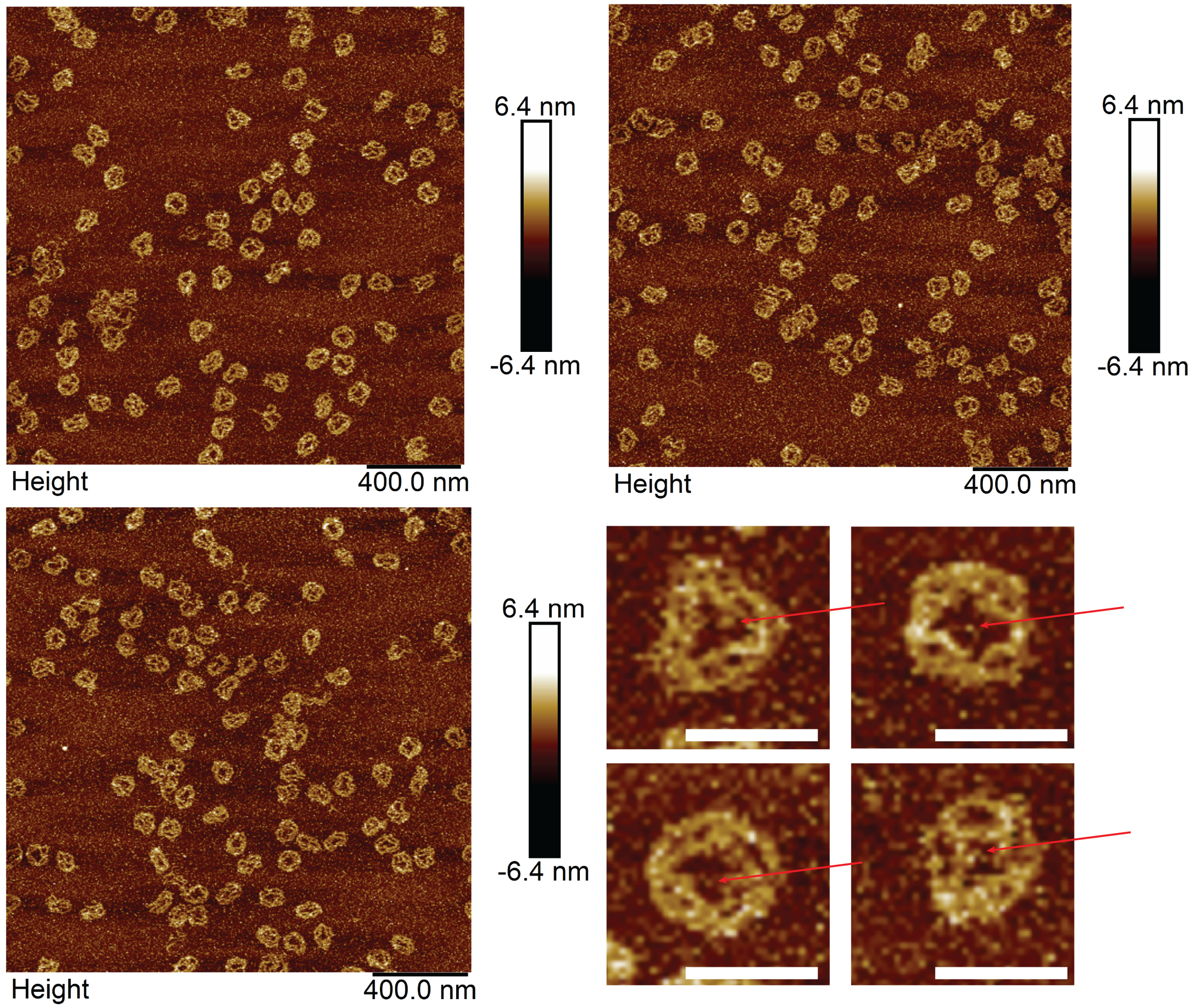

Figure S21: Tetrahedron binding to neutral ring. Zoomed-in scale bar = 100 nm. Red arrow pointing to possible nanostructure attached to the ring.

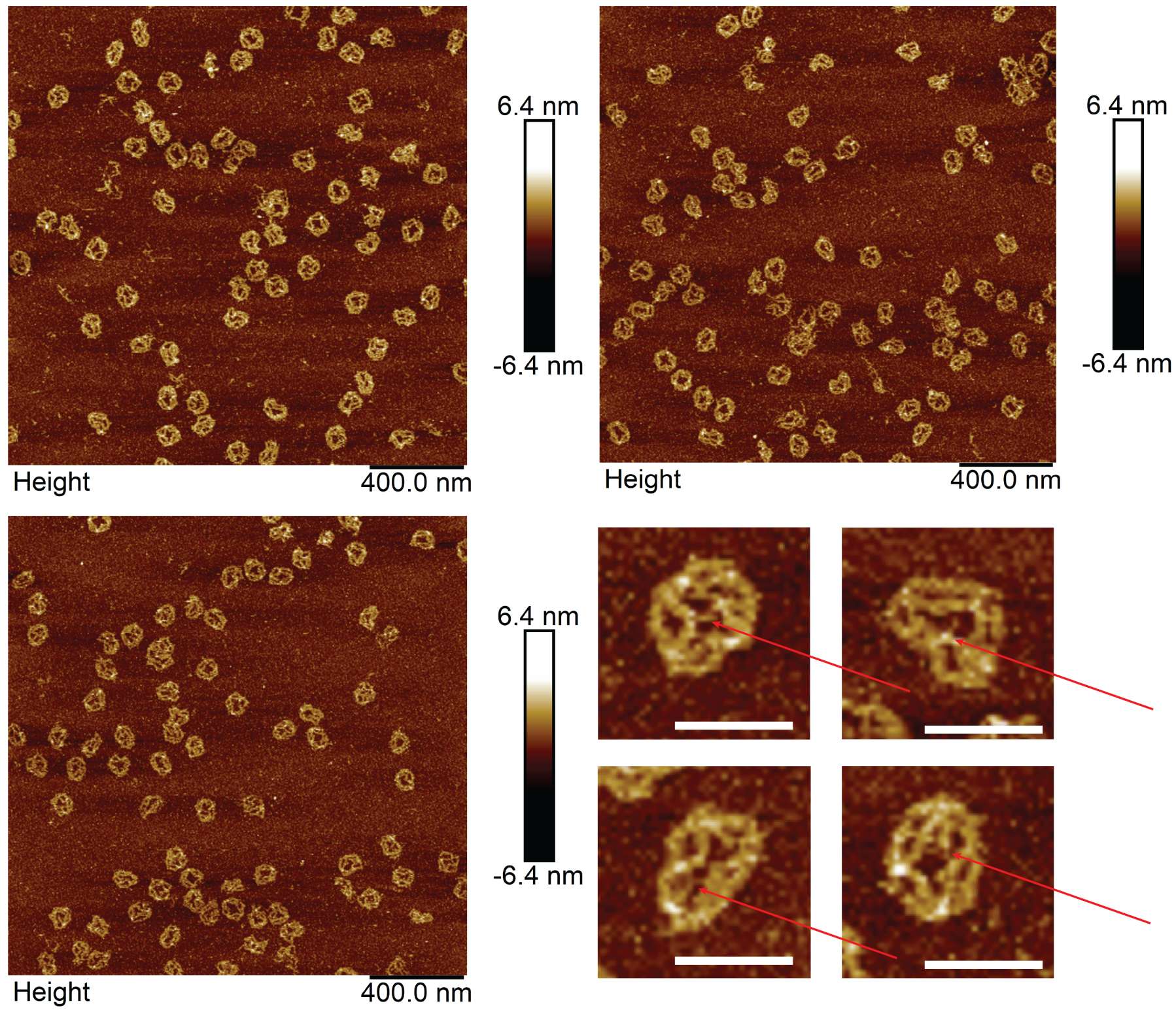

Figure S22: xTile binding to neutral ring. Zoomed-in scale bar = 100 nm. Red arrow pointing to possible nanostructure attached to the ring.

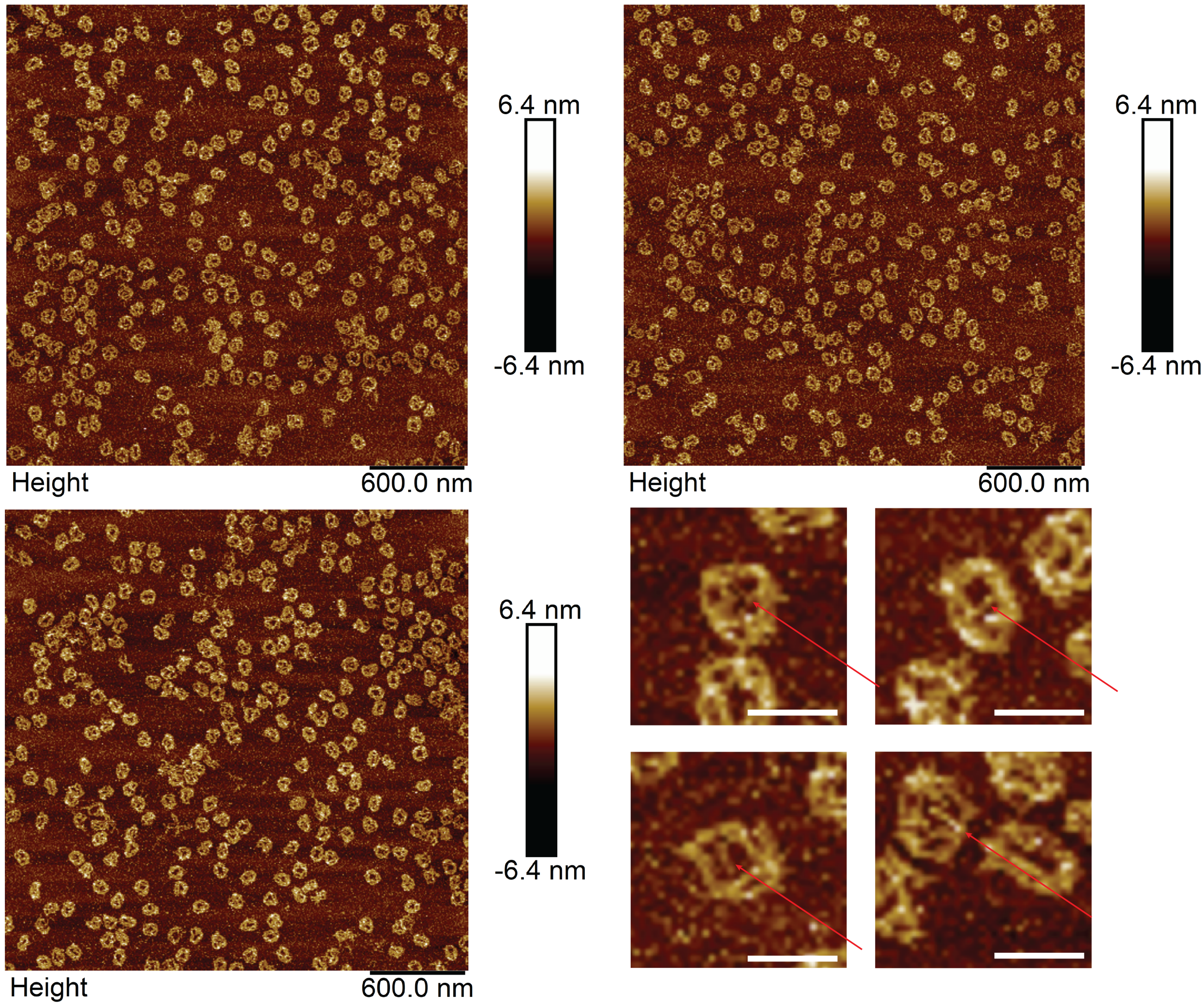

Figure S23: Needle binding to neutral ring. Zoomed-in scale bar = 100 nm. Red arrow pointing to possible nanostructure attached to the ring.

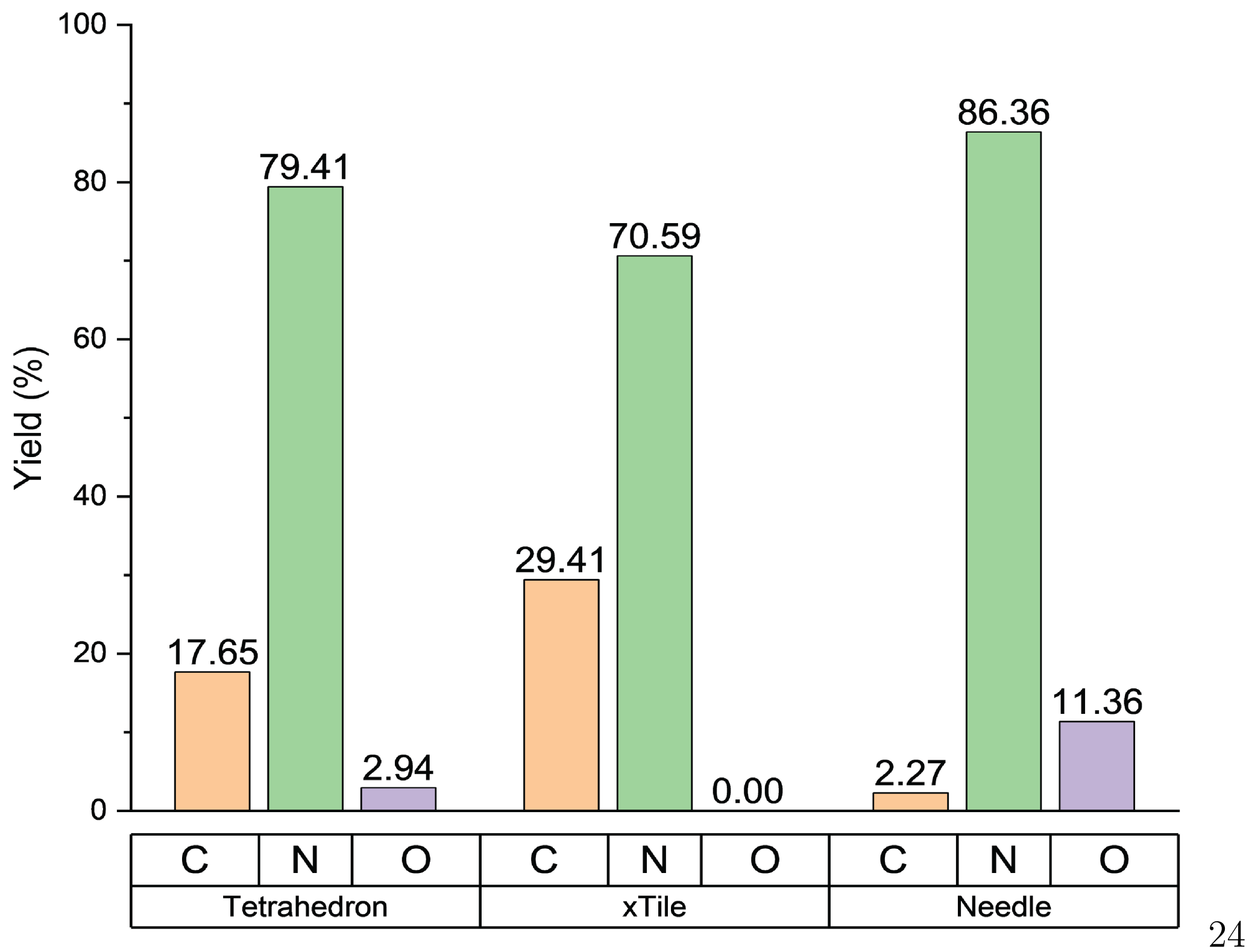

Figure S24: Ring conformation yield of the neutral ring binding the DNA nanostructures looking at the tetrahedron (N = 68), xTile (N = 85), and needle (N = 88). The closed (C), neutral (N), and open (O) percentages were determined.

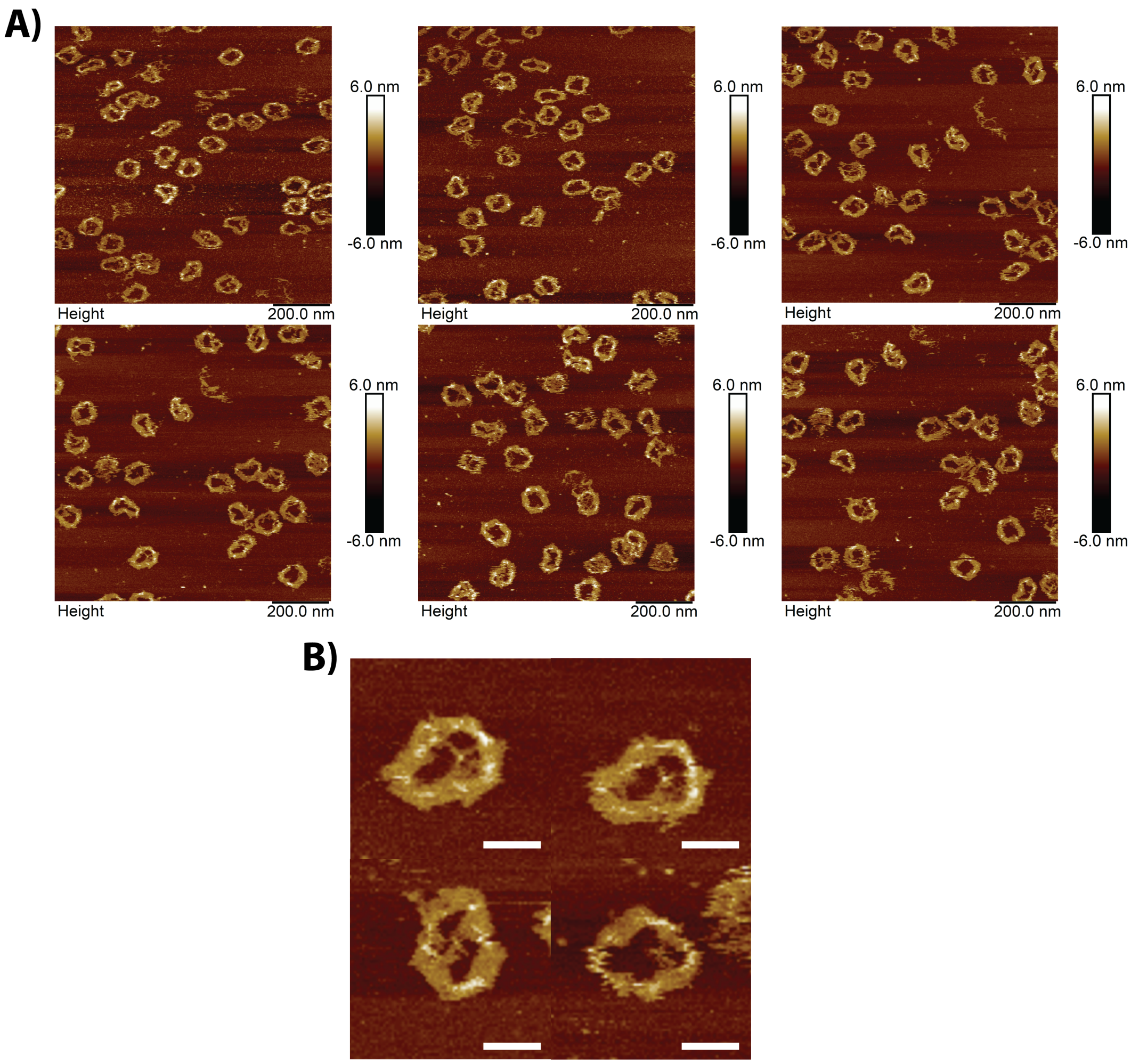

24

Figure S25: AFM of xTile binding to open ring. A) Overview of AFM. B) Zoomed-in AFM. Scale bar = 50 nm.

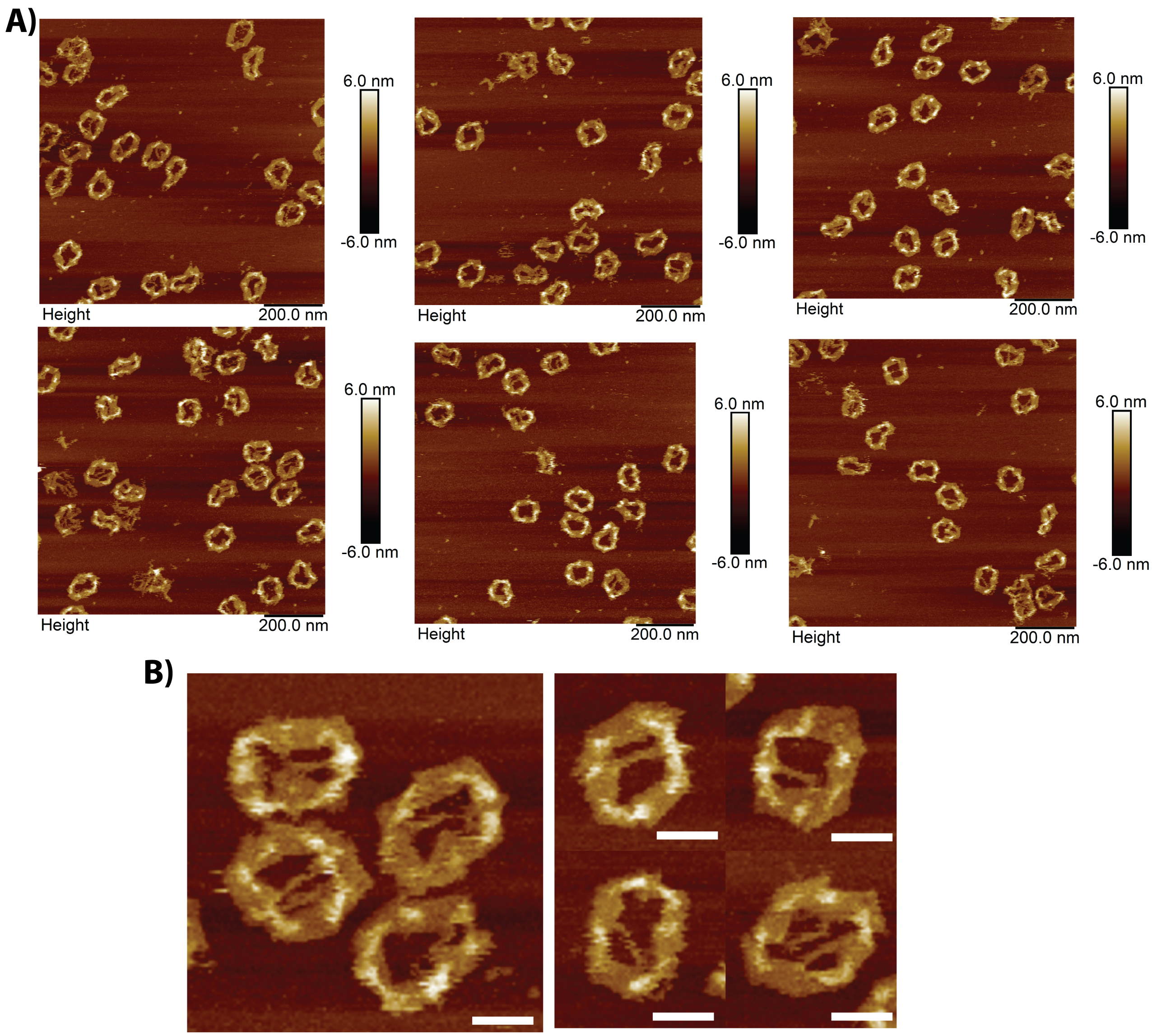

24

Figure S26: AFM of needle binding to open ring. A) Overview of AFM. B) Zoomed-in AFM. Scale bar = 50 nm.

Sequences

Neutral DNA Origami Ring:

Table S1:

Strand	Sequence
	**Body**
1	TTTTCTATCTTACCGAAGCCCTT
2	CGGCCTTGGAAAAATCTACGTTAATAGGGAGCTAAACAGG
3	TTTTGCATGTAGAAACCAATCAA
4	AGGGTCGGTTTGTAAACCGAACTGAATCAATA
5	TATTTTAGTAAATCCAATCGCAAGAAGAAGAGTC
6	TAATCGGCAAAATAATATCCCATCCTAATTTACGATTTT
7	TTTTCCGGCTTAGGTTGGGTTAT
8	CCGCCAGCATGGTCATAGCTACCAGTCAGGAC
9	TTTAAATTGTAAATCGTAAAACTAGCATTAATT
10	ACAATAGAACAATAAACAACATGTTACTAATA
11	CCGCCACCATAAGTGCCGTCGAGAGGGTTGATA
12	TTTTATTACCTTTTTTAATGTAACAATTTCATTTGATTTT
13	GAACGGTACGCCACTGACGAGAAACACCAGAA
14	GGCAACATATAAAAGAAACGCAAGAAATT
15	TTTTGCGCCAAAGACAAAAGG
16	CGGGAGAGATTCGCCTGATTGCTAAAACATAGC
17	GCGACATTCATCAATAGAAAATTCATATGGTTTACCATTTT
18	CAGAGAGATAAGGAAACCGAGGAAACGCAATAAT
19	GAGAGGCTTTTGCAAAAGTTTT
20	GCAATACTACCCTTCTGACCTGAAAGCGTA
21	ATTTAGACAAAATTAATTACATTGAAACAGTA
22	TCTGGTCAGTTGGCAAATCAA
23	TTGTTCCAGTTTGGAACAAGAG
24	AATCACCGGAACCAGAGAACCGCC
25	TTTTGTGATAAATAAGGCGTTA
26	CACAAACATTTAAATGCAATGCCTCAGTAGGGCTTAATTGAGAATCGCC
27	GGCCAGTATGAGTGAGCTAACTCTTAAGCCCAATAATAAAAGTAAGC
28	TTTTTCCGCGACCTGCTCCAATAAATTGTGTCGAAATTTT
29	TATTAGTCCCACCAGCAGAAGATAGAGGCGGTCAGTATTAGAACCGG
30	CAATTCTCAGCTAATGCAATAAATCCAAGAAC
31	TTTAAGAAGAGCAAGAAACAATGAAATAGCAATAGTTTT
32	AGAAACCACCAGAAGGAGCGGATTTT

Continued on next page

Table S1: (Continued)

33	AATAGTGAATTTATCAAAATCATATGTAAAT
34	TCACACGACCAGTAATAAAAGGGCAAACTAT
35	CAAACAAGGCCGCCACCCTCAGAACCCGGAGACAGTCAAA
36	AACCGCCAGAGACCCTCACCC
37	TTGTTAAAATTCGCATTAAAGTCTGGAG
38	TTTTAAGTTTTGCCAGAGGGGGTAATAGTAACAAAATAGC
39	TATCATTCCAAGAACGGGTATTGTTTATCA
40	AGATAGCCGAACAAAGTTACCAGAACCCACAA
41	ATCGCCTGTGTTACTTAGCCGGAAACAGATGA
42	TTTTAATTCGACAACTCGATTATCAGATGATGATTTAGAAGTATT
43	GCAAATCATTTTATCCTGAATCTCCCTTCACCGCCTGGCATATAAC
44	TGGAAAGCCAAGGATAAAAATTTTACAACGCCAACATGTAATTTAGGCA
45	ACGTGGCGAGAAAGGAGAGCCCCCG
46	ATAGGAACGCCATCAAAAATAATTCGTAGATGGG
47	ACGGTGTACACCTTGCTGAACCTCAAATAT
48	ATTGTTATCCGCGCTCGAATTC
49	GTCGCTATTAATTTCAATTACCTGAGCAATCTCCA
50	AAACGTAGAAAATACATAAAAACAGGGAAGC
51	GACTGGATAGCGTCCAATACTGCGATAACCCT
52	ACCAGTACAAACTACCTAAATTTAATGCCATGTACCGTAACTTTT
53	TTTTAGACAGCATCGGAACGAGGGTAGCAACTGCGGGAT
54	ACGGAACAAGGTAGAAAGATTCATTATAGTC
55	TTTTTCTAGAGGATCCCCCCTGGGGTGCCTAGCCAAGCTTGCATG
56	TTTTGCACTAAATCGGAAAGCGGTCACGCTGAGTTTTTTGGGGTC
57	CCTGCAGGTCGACTTTT
58	GATAGCTTAGATTAAGACGCTGCAAAGA
59	CCGACAATGACAACAAACGTAATGCCAC
60	ATAACTATAGGTCTGAGAGACTACCTTTTTAACCTTTTT
61	CATAAATCAATATATGTGAGTGAACAAACA
62	ATGATGAAATAACCTTGCTTCTGTAAATC
63	TAAGTATGGTTTAGTACCGCCACCAGGGATAG
64	TGACTATCAGTTGAGATTTAGGA
65	ACGCATAATGGAAGGGTTAGAACTAAAATATCTTTAGGAGCACTAACA
66	TCGGCAAAATCCCGAGCTTAATGGCGAAAATCCTGTTTGATTTT
67	AGTTGCAGTTTGCACCCAGCTACAAGATATA
68	TTCATCAGGATTGACCGTAATGGGCCTCAGGAAGATCGCAAACGTC
69	TCAAGAAAAGCTTGACGGGGTAATAATT
70	AATGGATTATTTACATTGGCAGCAATATTA

Continued on next page

Table S1: (Continued)

71	AATCGGTTGTACCAAAAATTTT
72	AGACGGTCCAAAGTACAACGGAGATTTGTATC
73	TTTTTGCCGGAAACCAGGCAAA
74	ACTAATAATTATACTTCTGAATAACCGATATATTCGGTCTCATGAGGAA
75	TACCTTATTACACATTTAATTGCTCCTTGAGTG
76	AGCTGATAAATTTAGCGTCAGACTG
77	CAAGCCCAAACACCGGAATCATAATTACT
78	ATTTTGCGGCTTTGCCCGAACGTTATGTCAATC
79	AGGAAGCCCGAAAGACTTCAAATAGACCGGAAG
80	TTTTCAGCAGCAAATGAA
81	CAGTAGCGACAGAATCAAGTTTGCCTTAATG
82	CGCATCGTCTGCCAGTTTGAGGGCCGTAAT
83	TTTTAAAAGCCCCAAAAACACCCGGTTGATAATCAGTTTT
84	CCGGAGTGCATAACCGAGGGTAGCTATTAACCA
85	ATTCACTTTAGCAGCTTAAAGGTGAAGATCGGT
86	GCCAAAAGAAATGCTTTAAACAGT
87	GCGCCATTCATTAGAGCCTTCCGGCACCGCTTCTGGTTTT
88	AGAAAAAGCCCCACCACCCTCATTTTCCTCAGAA
89	ACCAATGAAACCATCGATAGCAGCAGACGACGACAGTATCGGATAGGTCA
90	GCTCATTCAGTGAATAAGGCTTGCCGAATCCTGAGAAGTGTCCGTTGTA
91	CAAATCACGCGTAACCACCACACAGACGACGATAAAAACAATGTTTA
92	GCTGATGCTAATTTCATCTTCTGACAACGCC
93	GTAAGCGTCATACATGGCTTTTT
94	AAAAGAATAGCCCGAGATAGGGTTTTGATAAG
95	TTTTCTGTAGCTCAACA
96	AGAATACGTGCCATCACGCAAATTAATTTTATAATCAGTGAGCAAAGCT
97	TCCCTCAGAAGAATCGATGAACGGTAACGTTAATATT
98	TCAGACGATTGGCCTATTTCGGAACCTATTA
99	CAGGGCGGCCGCTACAGGGCGCGTAAGAGCAA
100	TTTTACTGAGTTTCGTC
101	TTAGCTATATTTTCATTTGGGGCGCGAGCTGAAAAGGTGGCAT
102	GCGTTTGCCATCTTTTCATAATCAA
103	TAACAGTACTTTCCAGACGTTAGTAAAT
104	TTTTTGGTGGTTCCGAAA
105	AGGTCATCAACAGGTCAGGATTAAGATTAAG
106	TGTAGCATTCCACAGACAGCCCTCATCAAATA
107	AAAAAAAGGCGAAATTGCGTAGAT
108	ATTTGAGGGCAATTCATCAATATAGTTAAAG

Continued on next page

Table S1: (Continued)

109	AAAGGAGCGGGCGCTTTT
110	GCGCCTAAACCAAGTACCGCACTCATCGAGA
111	TTTTATTATCATCATATTCCTGTATTAAATCAACAA
112	CAACATACGAGCCGTTTT
113	CAAACGGCACATTAAATGTGAGCGAGTAACAACCCTTTT
114	TATAAACAGTTAATGCCCCCTGCCTTGATATT
115	AGGCATAGTACTATGGTTGCTTTGCGTCAAAGGGCGAAAAACCGTCTAT
116	TTAAAACCGGCCACCAGTTTGAGTAACATTATC
117	GGAAGGGCTTATCACCGTCACCGAAAGGTAAA
118	TAAGAGAATATAATTTT
119	GTTTCCATTAAACGGGTAAAATCCATCGCCC
120	GGGGTTTTGCTCAGTACCAGGCGGCTCAG
121	TGTTTGGGATTAGAGCCGTCAATAGATAATAC
122	AACGGAATGAGGGTAATTGAGCGCCCAGTCG
123	TCAGAGTGTTTAGTATCATATGCGTTATA
124	CTTTAATTATCAGCTTGCTTTCTTTGAC
125	CTCATTATGTTTCCTGTGTGAA
126	TTTTCTACATTTTGACGCTCACGCTCATGGAAATACTTTT
127	AGTTGATTCCCAATTCTGCGAACGAGGGCAACAGCTGATTGTACCAACG
128	GTTGGGAACTGGTAATATCCAGAAATTCACCAG
129	GCCGCTTTGGCTACAGAGGCTTTGAGGACTAA
130	ATATGTACGGAAGATTGTATAAGCAAATA
131	ATGAAAATACAGAGAGAATAACATACATAAAGGT
132	AGAAGCAAAGCGGATTGCATCAAAAGAGAG
133	TTTTGACCTTCATCAAG
134	AGACTTTACAAACTTTT
135	TTTTGTCGGATTCTCCGTGGGAA
136	GAGGTGCCGTAAATTTT
137	CGAACGAATTTAATGCGCGAACTGATAGCCCTAAATTTT
138	GACGACGTAAGTCCTGAACAAGATGTCTTTCCT
139	TTTTGAAGCATAAAGTGTAAAGGGGTACCGATCACAATTCCACA
140	ACGCGTAACGAACAAAGAAAACTTTTTAGTTAG
141	TTTTTAGGGCGCTGGCAAGTGTCCCTAAAGGAGGGAAGAAAGCG
142	TTTTGCTTCAAAGCGAACCATCGCGTTTTAATTCGATTTT
143	TCAGAGCCACCCCGCCACCAGAACCACGGATTAGC
144	CACTATCGAATCGTCATAAATATTCATTGAATCCCCCTCGAATTACG
145	AGTTTCAGCGGAGTGAGAATAGAAAGGAACAA
146	TTTTCACGTTGAAAAAAGAAG

Continued on next page

Table S1: (Continued)

147	CGTCACCCTCAGCAGCGAATTTT
148	TTTTTAGCACCATTACC
149	GAGTAGAGGTAAACAAAAGAGTCTGTGCACAGACAATATTTTTGAATGGC
150	ATATTTATAGAACCCTCATATATAATAAATCCTCATTAAAGTGCCCG
151	TTTTAGTACCGACAAAAGCTTTTGCGGGAGATTTCGAGCCAGTAA
152	TGTTTTAAATATGCGTTTGCCCCAGCATGCTGAATATAATGTTTT
153	AATAAGAATAATAGGAACGTTTGAAATACCGACCGTTTTT
154	ACAAGCACATATTATTTATCCCAGAAACGA
155	AAATCTAAAGCATCAGACCAGGCAGTGCCACGCTGAGAGCTTTT
156	AACGGGGTCAGTGCCTTGAGTAACAGCCAGAA
157	TTACCTTATGCGATTTTAAGAACTGG
158	ATTAGCAAGGCCGGACTCCAGCCAGCTAGCAAAATCACCAGTTTT
159	TTTTCATTATGACCCTGTAATAGTAAAGTAAATAAAGCTA
160	TTTTTTGTTTAGAGGCGGTTTGCGT
161	GGAAACCTGAAGGCGATTAAGTTGGGTAACGCCA
162	CTAACGAGGAATCATTACCGCGCCCAATAGCAA
163	ATTTGGGAGCCATTCAGGCTGCGCAACTGTTG
164	CCCCAGCGATTATACCAAGCGCGAAAAATCATA
165	ATATTCATTACCCAAATCAACGTAAGCCACC
166	TATTGACGAGACACCACGGAATAAGTTTATT
167	CGTTGGTGCGTCTGGCCTTCCTGTAGCCAGCT
168	TTGTCACAAACCGATTGAGGGAGGGCTTGAGCC
169	GAATTGAGACATTAATTGCGTTGCCCCAGTCACGACGTTGTAAAACGAC
170	TTGCTATCAAGCGGTCCACGCTGAACTAAA
171	CAAACTCTTTTGCGGATGGCTTATTATAAATC
172	CGTAACGATCTAAAGTTTTGTCGTCCTTTTACAT
173	CGTTTACCCCGCCGCGCTTAATGCATGGCCCACTACGTGAACCATCACC
174	GTAATCCATTGCAACAGGAAAAAATCGTCTGA
175	GTAGTAGCATTAAAGCCTCAGAGCTTCTGTCCA
176	AGACTTTTGCTGAGGCTTGCAGGGAATCCTGAT
177	TTTTATCACCGTACTCAGGAAGCCCGGAATAGGTGTTTTT
178	GAGGCATAGCCTTTATTTCAACGGCAGTCTCTGAATTTATAAGTTTT
179	TTTTACATCGCCATTAAAAATAC
180	TTTTTTTGATGATACAGGAGTGTACTGGTAACCGTTCCA
181	TTTTCTTGCGGGAGGTTTTGAAG
182	GTACGGTGTCTGGAAGTTTCATTCCCCTGAGAG
183	CAAACCCTCACCAACTTTGAAAGAGGCGAGGCGC
184	CTAAAGGAATTGCGAAAAAGCCGGCGA

Continued on next page

Table S1: (Continued)

185	CCTTAAATCGCGAGGCGTTTTAGCGAACCTCCCGATTTT
186	AGTAATCTTGACAAACACCGCCTGCAACGCATAGGCTGGCTTTTT
187	GCGGGCCTCTTCGCTATTACG
188	GAAGGCTTATCCGGTATTCTAAGAACAAGATTAG
189	GGGTTTTGCTCACTGCCCGCTTTTAATAT
	**Turn**
190	TCAGAAAACGAGAATATAAATCAAAAATCAGGTCTTTACCC
191	AAGGATTACACCAGAGCCGCCGCCAGCATTGACAGTTGAGGCAGG
192	CAGTTGAAAGGAATTGTTTTAGGAAGGTTATCCTACCAT
193	TCCACTATTAAAGAACTTTTGTGGACTCCAAACGAGCAC
194	CAAATTCTTACCAGTATAAAGTTTTCCAACGCTCAAGAGTAATG
195	GTATAACGTGTTCTCGTTAGAATCAGAGCAAACGAACTA
196	TGCCTGAGATTTTTGTTAAATTTTTCAGCTCATTTTTTTTTGAG
197	CCAAGTTACAATTCGCAGAGGCGA
198	AGGCCGATTAGATTTTAGACAG
199	TCACCATCAAATATTCAACCGTTCT
200	ATACCACATTTAATGCAGATACATAAC
201	TTCTGAAACATGAATTAAGAGGCTGAGACTCCTCAAGAG
202	GAACTGGCATGATTAAGACTCCTACGCAGTATGTTAGC
203	TACGAAGGCACCAACCTAAAAAGAGGCAAAAGAATACACTAAAA
204	AGATCTACAATTATCAGGTCAT
205	GCATTAGACGGGATAACTGAACACCCTGAACAAAGTCAACCCAAAA
206	GAATTTTCTGTAATTTTGCTAAACAACTTTCAAC
207	TAAACAGCAGCCGTTTTTATTTTTTTTCATCGTAGCGTCTTTC
208	TAGTAATAACTTTTGCCTGAGT
209	CCAGCTGGCGAAAGGGTTTTGGATGTGCTGCTCGTGCCA
210	AGAAGAACTACATTCTGGCCATTTTACAGAGATAGATCTTTGAT
211	ATTGGGCGCCTGGTTTTTCTTTTCACCAGTGAGACGTAGATTT
212	ATTATTCATTAATTTTCCCTTTTTTAGAATCCTTGTTGAATA
213	ATCAAAATTATTTCGTAAAACAGAAATAAATCCAAAAGGAGC
214	CGAGTAGTAAATCTTGAGATGGTTTAATTTCAACTTTAATCATTGTGAA
215	TTTCAGGTTTTCAGATGAATATACAG
216	GCTGCATTAATTCGGCCAACGCGCGGGGAACGTCAAAA
217	TGTAGGTAAATTAAAAGGGTGAGAAAGGCGCCACCC
218	CAGAGCCTAATTCAGTTACAAAA
219	CACTCATCGAGGTGAATTTCTTAAACAGCTTGATATAGTTGCG
220	TAGCGCGTTTTCATCGTTTCGGTCATAGCCCCCTTATTA
221	AGTTTGACCATTAGATACATTTCATGGTCAATAACCTGT

Closed and Open Conformations:

Table S2:

Strand	Sequence
	**Closed-Locked**
1	TTTTGTGATAAATAAGGCGTTAAAATGTGAGCGAGTAACAACCCTTTT
2	TTTTTGGTGGTTCCGAAATCGGCAGCGAACTGATAGCCCTAAATTTT
3	CCTCAAATATCAATCAATAGTGAATTTA
4	TGCGCAACTGTTGGGAAGGCTTATCCG
5	ATCATAATTACTAGAGCCTTCCTGTAGCCAGCT
6	TTTTCAGCAGCAAATGAAAAATCTAGAGACTACCTTTTTAACCTTTTT
7	TTTTTGCCGGAAACCAGGCAAAGCGTTTTAGCGAACCTCCCGATTTT
8	AAAAGAATAGCCCGAGAATATTTTTGAATGGC
	**Open-Locked**
1	TTTTACTGAGTTTCGTCACCAGTAGTATTAGACTTTACAAACTTTT
2	GTAGCATTCCACAGACCCGTCAATAGATAATAC
3	ACCAATGAAACCATCGAAACATGTAATTTAGGCA
4	TTTTTAGCACCATTACCATTAGCCAGTAATAAGAGAATATAATTTT
5	TTTTGACCTTCATCAAGAGTAATGGGGTCGAGGTGCCGTAAATTTT
6	CCACGCTGAACTAAAGTACGGTGTCTGGAAGTCGACGTTGTAAAAC GACGGCCAGTATGAGTGAGC
7	TTTTCTGTAGCTCAACATGTTTTTGCATGCCTGCAGGTCGACTTTT
8	CAGTATTAGAACCGGATATTCATTACCCAAATCTACGTGAACCATCAC CCAAATCACGCGTAAC
	**Closed-Toehold**
1	TACTGCTTTTTGCCGGAAACCAGGCAAAGCGTTTTAGCGAACCTCCCGATTTT
2	CAGGCGTGCGCAACTGTTGGGAAGGCTTATCCG
3	CTCCAATTTTTGGTGGTTCCGAAATCGGCAGCGAACTGATAGCCCTAAATTTT
4	GTATCGAAAAGAATAGCCCGAGAATATTTTTGAATGGC
5	AAGGAGTTTTCAGCAGCAAATGAAAAATCTAGAGACTACCTTTTTAACCTTTTT
6	GCCCTACCTCAAATATCAATCAATAGTGAATTTA
7	CCTTAGTTTTGTGATAAATAAGGCGTTAAAATGTGAGCGAGTAACAACCCTTTT
8	CAGACAATCATAATTACTAGAGCCTTCCTGTAGCCAGCT
	**Open-Toehold**
1	TTTTACTGAGTTTCGTCACCAGTAGTATTAGACTTTACAAACTTTTAGCCAC
2	TTTTCTGTAGCTCAACATGTTTTTGCATGCCTGCAGGTCGACTTTTCTCCGG
3	CCACGCTGAACTAAAGTACGGTGTCTGGAAGTCGACGTTGTAAAACGACGG CCAGTATGAGTGAGCTGTACG
4	GTAGCATTCCACAGACCCGTCAATAGATAATACGTACGC
5	TTTTGACCTTCATCAAGAGTAATGGGGTCGAGGTGCCGTAAATTTTGGACGT

Continued on next page

Table S2: (Continued)

6	CAGTATTAGAACCGGATATTCATTACCCAAATCTACGTGAACCATCACCCAAAT CACGCGTAACGACGAG
7	ACCAATGAAACCATCGAAACATGTAATTTAGGCAGCTTCG
8	TTTTTAGCACCATTACCATTAGCCAGTAATAAGAGAATATAATTTTTTGGCG
	**Closed-Toehold Invaders**
1	AAAATCGGGAGGTTCGCTAAAACGCTTTGCCTGGTTTCCGGCAAAAAGCAGTA
2	CGGATAAGCCTTCCCAACAGTTGCGCACGCCTG
3	AAAATTTAGGGCTATCAGTTCGCTGCCGATTTCGGAACCACCAAAAATTGGAG
4	GCCATTCAAAAATATTCTCGGGCTATTCTTTTCGATAC
5	AAAAAGGTTAAAAAGGTAGTCTCTAGATTTTTCATTTGCTGCTGAAAACTCCTT
6	TAAATTCACTATTGATTGATATTTGAGGTAGGGC
7	AAAAGGGTTGTTACTCGCTCACATTTTAACGCCTTATTTATCACAAAACTAAGG
8	AGCTGGCTACAGGAAGGCTCTAGTAATTATGATTGTCTG
	**Open-Toehold Invaders**
1	GTGGCTAAAAGTTTGTAAAGTCTAATACTACTGGTGACGAAACTCAGTAAAA
2	CCGGAGAAAAGTCGACCTGCAGGCATGCAAAAACATGTTGAGCTACAGAAAA
3	CGTACAGCTCACTCATACTGGCCGTCGTTTTACAACGTCGACTTCCAGACACC GTACTTTAGTTCAGCGTGG
4	GCGTACGTATTATCTATTGACGGGTCTGTGGAATGCTAC
5	ACGTCCAAAATTTACGGCACCTCGACCCCATTACTCTTGATGAAGGTCAAAA
6	CTCGTCGTTACGCGTGATTTGGGTGATGGTTCACGTAGATTTGGGTAATGAA TATCCGGTTCTAATACTG
7	CGAAGCTGCCTAAATTACATGTTTCGATGGTTTCATTGGT
8	CGCCAAAAAATTATATTCTCTTATTACTGGCTAATGGTAATGGTGCTAAAAA

Capturing:

Table S3:

**Strand**	**Sequence**
	**Gold Nanoparticle Capturing Strands**
1	TGTTTTAAATATGCGTTTGCCCCAGCATGCTGTCAATC
2	TTTTCTTGCGGGAGGTTTTGAAGGCTGTCAATC
3	AGTAATCTTGACAAACACCGCCTGCAACGCTGTCAATC
4	TTTTACATCGCCATTAAAAATACGCTGTCAATC
5	ACCAGTACAAACTACCTAAATTTAATGCGCTGTCAATC
6	TTTTCCGGCTTAGGTTGGGTTATGCTGTCAATC
7	ATTAGCAAGGCCGGACTCCAGCCAGCTAGCTGTCAATC
8	TTTTGTCGGATTCTCCGTGGGAAGCTGTCAATC

Continued on next page

Table S3: (Continued)

	**Gold Nanoparticle Thiol-Modified Strand**
1	/5ThioMC6-D/TTTTTTTTTTGATTGACAGC
	**DNA Nanostructure-Origami Glue Strand**
1	TAACAGTCTACAGCCATGGTCGGATTGACAGC

Nanostructures:

Table S4:

Strand	Sequence
	**Cy5 Modified Strand**
1	/Cy5/ATAAGCTATAAATGTTGAGGTT
	**Tetrahedron**
1	CGACCATGGCTGTAGACTGTTATTTTTACATTCCTAAGTCTGAAA CATTACAGCTTGCTACACGAGAAGAGCCGCCATAGTA
2	CGACCATGGCTGTAGACTGTTATTTTTTATCACCAGGCAGTTGAC AGTGTAGCAAGCTGTAATAGTACGCTCCCAGGTTATG
3	CGACCATGGCTGTAGACTGTTATTTTTTCAACTGCCTGGTGATAA AACGACACTACGTGGGAATCTACTATGGCGGCTCTTC
4	AACCTCAACATTTATAGCTTATTTTTTTTCAGACTTAGGAATGTGC TTCCCACGTAGTGTCGTTTCATAACCTGGGAGCGTA
	**xTile**
1	TGCATCGGTGGCAGTACG
2	GATTGTCACCATGATG
3	GTGTTGTCACCATGATGG
4	GCCGTAGTGGCGCAGT
5	GCAACCCACCACTGCTCAGTCAGAGTGGATGGAGGCTGGATC
6	ACTCCTATGCCGTTGAGCAACATAGGAGGGAGGACTTTATCA
7	GCGATAGTGGCCTGAGCAGAAAGACACCAGCCTGAGAAACCC
8	ATACATCACCTCCGGAGATCGCACTCTCCATCCTGTTTCTCC
9	CGTACTGCCTGGGTTGCGGAGAAACCCATCCTGTCTTTCTGCTCAGGCCTGACAACAC
10	ACTGCGCCTGATGTATGATCCAGCCTAGGATGGTCCTATGTTGCTCAACGGCTGACAATC
11	CCATCATGGACTATCGCTGATAAAGTAGGCTGGACTCTGACTGAGCAGTGGACCGATGCA
12	CATCATGGATAGGAGTGGGTTTCTCCCTCCCAGAGTGCGATCTCCGGAGGACTACGGC
13	CGACCATGGCTGTAGACTGTTACGTACTGCCTGGGTTGCGGAGAAA CCCATCCTGTCTTTCTGCTCAGGCCTGACAACAC
14	CGACCATGGCTGTAGACTGTTAACTGCGCCTGATGTATGATCCAGCC TAGGATGGTCCTATGTTGCTCAACGGCTGACAATC
15	CGACCATGGCTGTAGACTGTTACCATCATGGACTATCGCTGATAAAGT AGGCTGGACTCTGACTGAGCAGTGGACCGATGCA

Continued on next page

Table S4: (Continued)

16	AACCTCAACATTTATAGCTTATCATCATGGATAGGAGTGGGTTTCTCCC TCCCAGAGTGCGATCTCCGGAGGACTACGGC

Needle Tile

1	CCGGGTGTGGGCAATACAGGTCCG
2	TGGTTTGTACGTGTGCCTGCTGACTG
3	CCAGAGCATCGGTGGCAGTAACGCTGTATTGTCACCATGATT
4	CCTGGGGCCAAGTGGGCGGGTCCCGTGGTTGGCACCAGCGAT
5	AGGTAGAAGACCACCGGGCAGGAATCTGGCTGGTGGAAGCCG
6	GCCCCAGGATCGCTGGACCGATGCTCTGGAATCATGGACACCCGG
7	GGGACCCGCCCTGGTCTTCTACCTCGGCTTCCTACAAACCA
8	TTTTTCAGTCAGCAGGCACACGACCAGCCAGATTCCTGCCCGGACTTG
9	CGGACCTGTATTGCCCTGACAATACAGCGTTACTGCCTGCCAACCAC
10	CGACCATGGCTGTAGACTGTTACGGACCTGTATTGCCCTGACAATACAGCG TTACTGCCTGCCAACCAC
11	CGACCATGGCTGTAGACTGTTACCGGGTGTGGGCAATACAGGTCCG
12	CGACCATGGCTGTAGACTGTTATGGTTTGTACGTGTGCCTGCTGACTG
13	AACCTCAACATTTATAGCTTATTTTTTCAGTCAGCAGGCACACGACCAGCC AGATTCCTGCCCGGACTTG
